# Dynamics and functional roles of splicing factor autoregulation

**DOI:** 10.1016/j.celrep.2022.110985

**Published:** 2022-06-21

**Authors:** Fangyuan Ding, Christina J. Su, KeHuan Kuo Edmonds, Guohao Liang, Michael B. Elowitz

**Affiliations:** 1 Division of Biology and Biological Engineering, California Institute of Technology, Pasadena, CA 91125, USA; 2 Howard Hughes Medical Institute; 3 Department of Biomedical Engineering, University of California, Irvine, Irvine, CA 92697, USA; 4 Center for Synthetic Biology, Center for Complex Biological Systems, Chao Family Comprehensive Cancer Center, Department of Developmental and Cell Biology, and Department of Pharmaceutical Sciences, University of California, Irvine, Irvine, CA 92697, USA; 5 Present address: University of Illinois College of Medicine, Chicago, IL 60612, USA; 6 Lead contact

## Abstract

Non-core spliceosome components are essential, conserved regulators of alternative splicing. They provide concentration-dependent control of diverse pre-mRNAs. Many splicing factors direct unproductive splicing of their own pre-mRNAs through negative autoregulation. However, the impact of such feedback loops on splicing dynamics at the single-cell level remains unclear. Here, we developed a system to quantitatively analyze negative autoregulatory splicing dynamics by splicing factor SRSF1 in response to perturbations in single HEK293 cells. We show that negative autoregulatory splicing provides critical functions for gene regulation, establishing a ceiling of SRSF1 protein concentration, reducing cell-cell heterogeneity in SRSF1 levels, and buffering variation in transcription. Most important, it adapts SRSF1 splicing activity to variations in demand from other pre-mRNA substrates. A minimal mathematical model of autoregulatory splicing explains these experimentally observed features and provides values for effective biochemical parameters. These results reveal the unique functional roles that splicing negative autoregulation plays in homeostatically regulating transcriptional programs.

## In brief

Negative autoregulatory feedback is a prevalent feature of splice regulation. Ding et al. show that the splice regulator SRSF1 uses negative autoregulatory splicing to maintain constant levels independent of variations in its own transcription and the “load” of pre-mRNA transcripts. A minimal mathematical model can explain these capabilities.

## Graphical Abstract

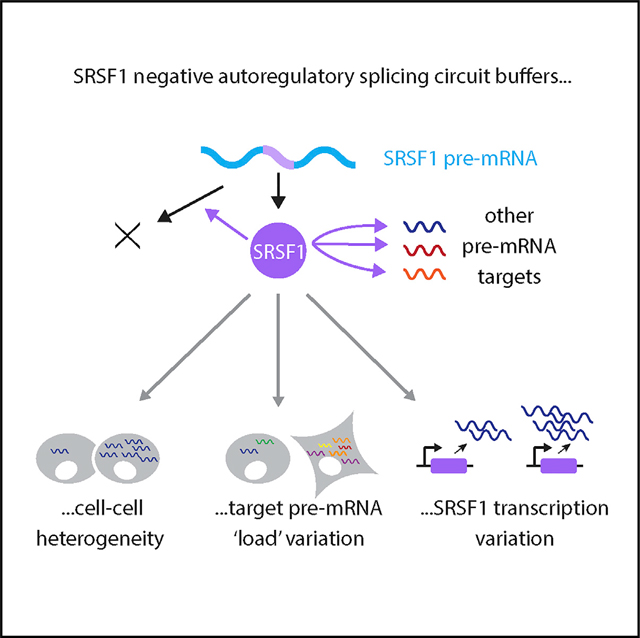

## INTRODUCTION

More than 90% of a typical eukaryotic genome undergoes alternative splicing, producing multiple mRNA isoforms and expanding proteome diversity (Merkin et al., 2012; Barbosa-Morais et al., 2012; Pan et al., 2008; Wang et al., 2008; Nilsen and Graveley, 2010; Ule and Blencowe, 2019; Chaudhary et al., 2019; Liu et al., 2017). Missplicing can cause diverse physiological effects and lead to disease (Scotti and Swanson, 2016; Faustino and Cooper, 2003; Kalsotra and Cooper, 2011; Urbanski et al., 2018; Bonnal et al., 2020; Thomas et al., 2020). Alternative splicing is controlled by many distinct components (Black, 2003; Lee and Rio, 2015; Wilkinson et al., 2020), including non-core spliceosome components (Jangi and Sharp, 2014), the splicing code (Barash et al., 2010; Culler et al., 2010), RNA secondary structures (McManus and Graveley, 2011), RNA polymerase speed (Fong et al., 2014), and epigenetic regulation (Luco et al., 2011).

Of these regulators, non-core spliceosome components play a unique role by modulating splicing activity in a concentration-dependent manner, and maintaining a stable milieu of their concentration (i.e., splicing activity) is critical (Black, 2003; Wang and Burge, 2008; Shin and Manley, 2004; Gehring and Roignant, 2021). Most splicing regulators fall into two main, conserved families, serine-arginine rich (SR) proteins and heterogeneous nuclear ribonucleoproteins (hnRNPs), and are found across diverse tissue types and species (Manley et al., 1996; Dreyfuss et al., 1993; Zahler et al., 1992; Baralle and Giudice, 2017), ranging from *Schizosaccharomyces pombe* (Shepard and Hertel, 2009) to *Arabidopsis* (Kalyna et al., 2006). SR or hnRNP proteins modulate alternative splicing of large and diverse sets of target genes (Long and Caceres, 2009; Zhou and Fu, 2013; Wang and Manley, 1995; Leclair et al., 2020), and are implicated in diverse disease processes (Geuens et al., 2016; Anczuków and Krainer, 2016; Bonnal et al., 2020). Maintaining splicing factor homeostasis is thus critical for cellular function.

The control of splicing factor level is commonly achieved through negative autoregulatory splicing (Kalsotra and Cooper, 2011; Lareau et al., 2007; Ni et al., 2007). Specifically, splicing factors alternatively splice their own pre-mRNA to unproductive isoforms, either containing premature termination codons (Wollerton et al., 2004) or introducing new junctions in the 3′ untranslated region (UTR) (Sureau et al., 2001), to trigger degradation by RNA surveillance pathways (Maquat, 2004). The overexpression of splicing factors promotes unproductive splicing (Sureau et al., 2001), leading to negative autoregulation. Previous work investigated many aspects of negative autoregulation, including associated highly or ultra-conserved sequence motifs (Lareau et al., 2007; Ni et al., 2007) and related nonsense-mediated mRNA decay (NMD) triggered by this regulatory mode (Hug et al., 2016; Ni et al., 2007). Nevertheless, the basic question of how autoregulation plays out dynamically at the single-cell level remains unclear.

**Figure 1.**
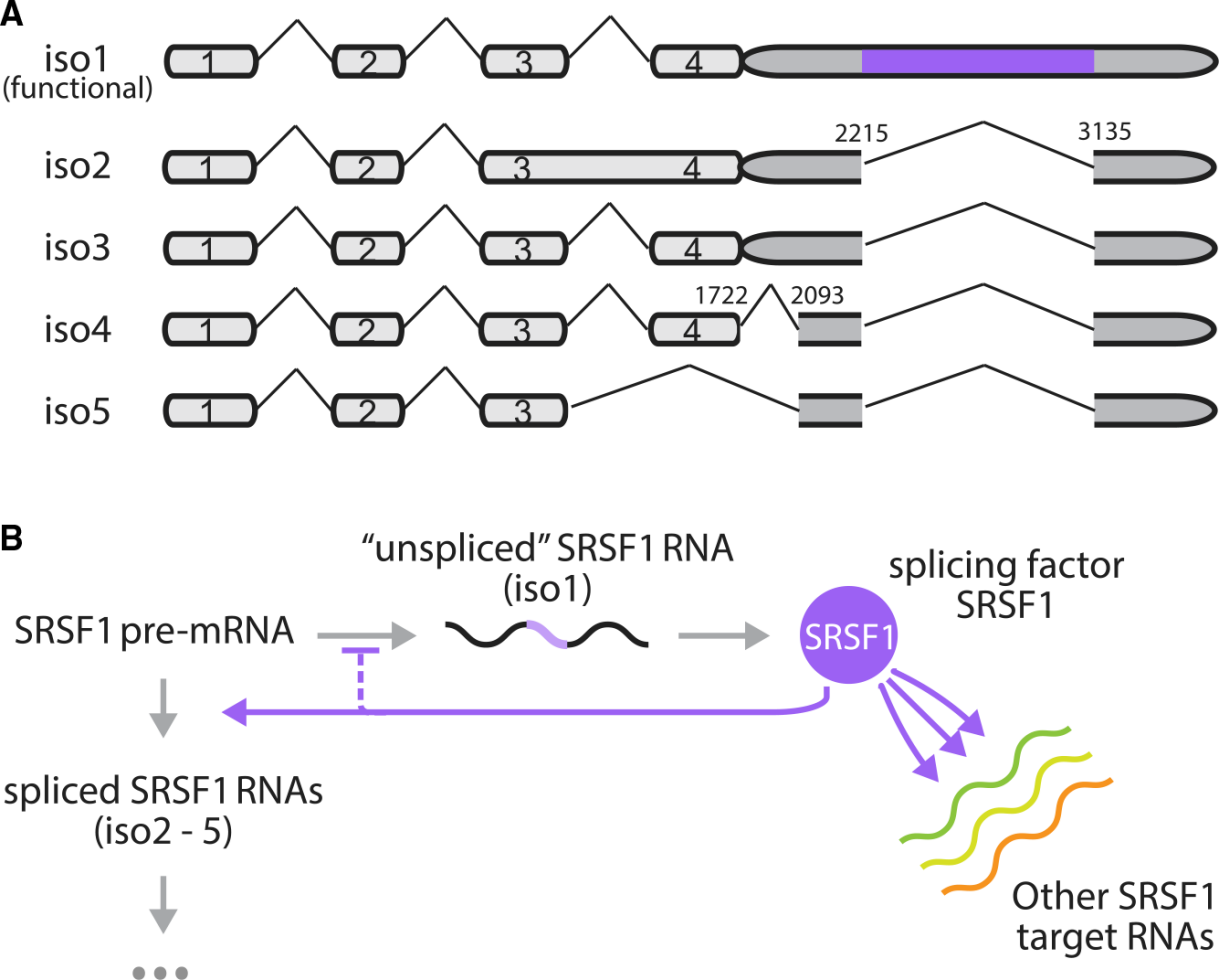
Splicing factors negatively autoregulate their own synthesis by promoting unproductive splicing of their own transcripts and also operate under variable loads of substrate pre-mRNA produced by other genes (A) Five isoforms of *SRSF1* were observed in HEK293 cells. Each pre-mRNA molecule can be spliced to remove introns in the 3′ UTR (without light purple isoforms, isoforms 2–−5) or left unspliced (with light purple isoforms, isoform 1). Intron removal can lead to degradation through RNA surveillance pathways, while transcripts with retained introns in the 3′ UTR (isoform 1) are translated to produce more splicing factor SRSF1. (B) Feedback occurs when splicing factors enhance intron removal from their own pre-mRNA, thus negatively regulating their own expression. Apart from their own transcripts, splicing factors additionally act on transcripts produced by other genes. The relative abundance of substrate pre-mRNAs can affect the allocation of splicing factors among transcripts, thereby influencing the dynamics of splicing negative autoregulation.

In other contexts, negative autoregulatory transcriptional feedback is known to speed response times and promote robustness to perturbation (Becskei and Serrano, 2000; Elowitz et al., 2002; Rosenfeld et al., 2002; El-Samad, 2021). However, negative splicing regulation potentially has unique features compared to transcriptional feedback. For instance, rather than operating at a fixed number of DNA binding sites, splicing factors can operate at diverse “loads” of pre-mRNA substrates from their own and other target genes in the cell (Figure 1B). Due to the effects of stochastic gene expression (Raj and van Oudenaarden, 2008; Bohrer and Larson, 2021), total substrate amounts can vary between cell states or over time. This provokes the question of what role splicing autoregulation may play in enabling homeostatic control of splicing factor levels and accelerating responsiveness to changes in substrate.

To address these questions, we developed a system to dynamically and quantitatively investigate negative autoregulatory splicing at the single-cell level. We focused specifically on SRSF1, the protein product of the *serine/arginine-rich splicing factor 1* (*SRSF1*) gene, which is widely expressed in distinct cell types and regulates the splicing pattern of many important genes (Karni et al., 2007; Li and Manley, 2005; Anczuków et al., 2012; Sanford et al., 2009; Paz et al., 2021). Isoform 1 of the *SRSF1* transcript, retaining an intron in its 3′ UTR (Figure 1A), is functional (produces SRSF1 protein). We call this the “unspliced” isoform in Figure 1B because it retains a specific intron in its 3′ UTR region, although other introns between exons 1 and 4 of isoform 1 remain spliceable (Figure 1A). To differentiate them from isoform 1, we collectively refer to the other isoforms (Figure 1A iso 2–5) as “spliced” (Figure 1B). These other isoforms are unproductive (do not produce SRSF1 protein).

By tracking SRSF1 accumulation using time-lapse videos with a machine learning-based image analysis system and analyzing SRSF1 levels together with flow cytometry and qRT-PCR/PCR, we found that negative autoregulatory splicing can buffer SRSF1 concentration, achieve ~50% less cell-cell heterogeneity in both expression and response rate upon perturbation of its own pre-mRNA levels, and enable adaptation to total substrate load at both a single-cell level and across 53 human tissue types. We then demonstrated how negative splicing autoregulation can maintain its own dynamics and how it adapts to perturbation in a minimal model. Together, these results quantitatively explain the single-cell dynamics of negative splicing autoregulation and reveal its functional role and impact as a regulatory circuit.

## RESULTS

### SRSF1 maintains homeostasis through negative splicing feedback

**Figure 2.**
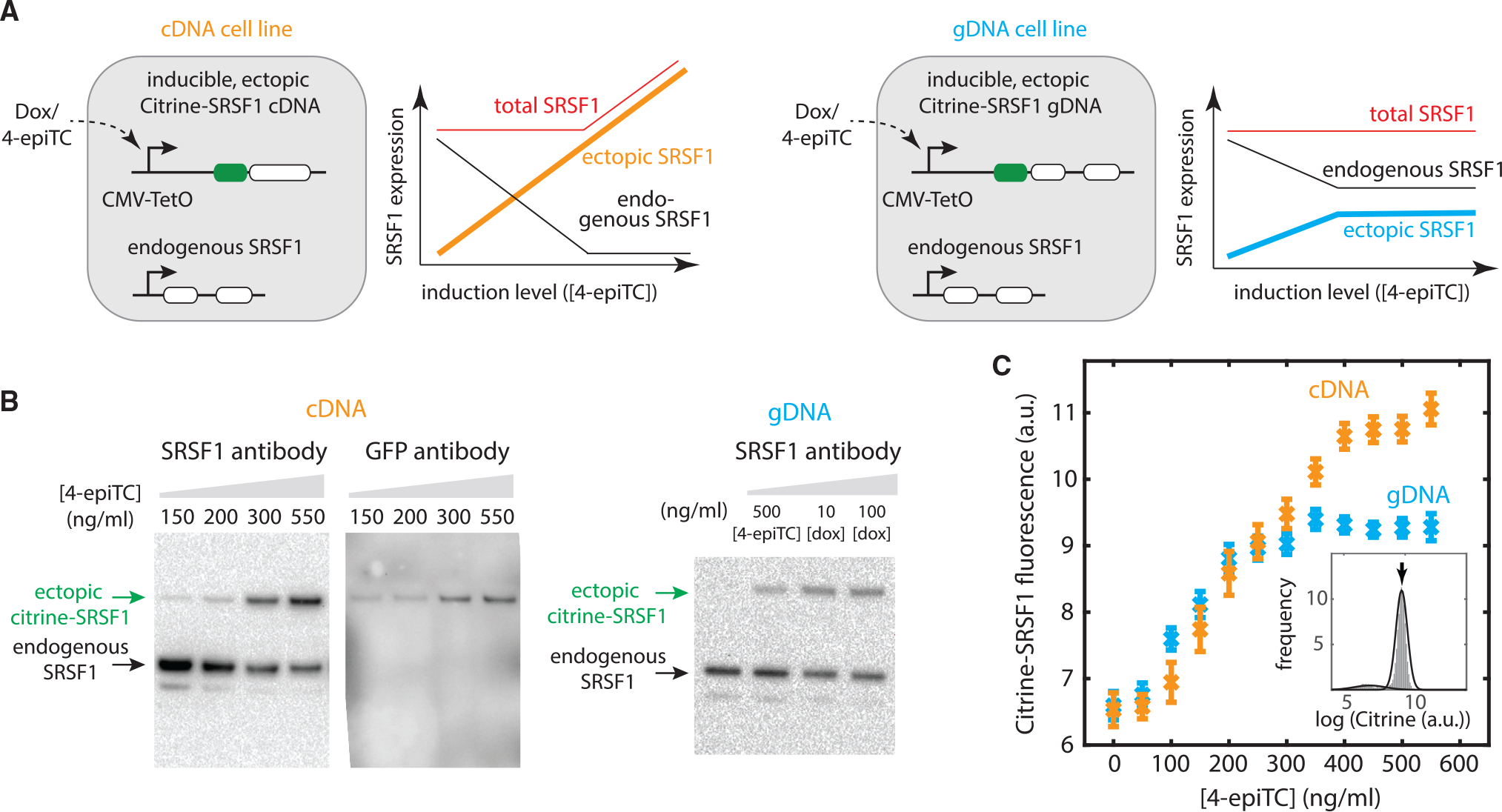
Negative splicing autoregulation establishes a ceiling for SRSF1 protein concentration in response to its own pre-mRNA substrate perturbations (A) We designed two cell lines: one transfected with a Citrine fused SRSF1 cDNA (i.e., unregulated, with no intron, shown in orange), the other transfected with a Citrine-fused genomic SRSF1 DNA (i.e., autoregulated, shown in blue), both under an inducible Tet-On CMV promoter and stably integrated into the fixed locus of Flp-In T-REx HEK293 cell lines (top). (Bottom) Expected outcomes (schematic): For the cDNA version, increasing ectopic SRSF1 protein level should downregulate endogenous *SRSF1* production via splicing feedback (black curve). When the endogenous copy saturates its ability to buffer *SRSF1* overexpression, the total SRSF1 level overshoots (red curve). By contrast, for the gDNA cells, due to the negative splicing autoregulation of both the ectopic copy (blue curve) and endogenous copy (black curve), the total SRSF1 level should remain constant (red curve), across a broader range of induction levels. (B) Western blot shows that the endogenous SRSF1 level decreases with the increasing expression of the ectopic copy. We induced cDNA cells at different 4-epiTC (an analog of doxycycline [dox], with weaker affinity) concentration for 24 h. Anti-SRSF1 antibody (ab133689) staining shows 2 bands: the top band indicates the ectopic copy with fused Citrine (verified by staining Citrine using anti-GFP monoclonal antibody [right]), the bottom band indicates the endogenous copy. Western blot of gDNA cells (induced at different 4-epiTC/dox concentration for 24 h) is also shown as a comparison. (C) Flow cytometry data shows that ectopic SRSF1 reaches a ceiling (blue curve) with negative splicing autoregulation (i.e., gDNA version), but not with the cDNA version. The 2 cell lines (A) were induced at different 4-epiTC concentration for >24 h and analyzed by flow cytometry. Mean expression levels were extracted from Gaussian fits (Figure S3) to represent the ectopic SRSF1 level. Error bars represent the standard error of the mean from 9 experimental replicates.

We set out to investigate negative autoregulatory splicing by engineering two HEK293 cell lines expressing the *SRSF1* gene with or without autoregulation. We site specifically and stably integrated a single ectopic copy of *SRSF1*, using either genomic sequence (guide DNA [gDNA], autoregulated) or cDNA sequence (unregulated), under the control of a doxycycline (dox)-inducible cytomegalovirus (CMV) promoter (Figure 2A, top). Because SRSF1 protein is essential for cellular physiology (Li and Manley, 2005; Wang and Manley, 1995), the endogenous genomic copy of *SRSF1* was left intact in both cell lines. To distinguish ectopic and endogenous *SRSF1*, we fused a fluorescent protein, Citrine, at the 5′ end of the ectopic copies (Figure 2A, top). The addition of Citrine did not affect *SRSF1* RNA or protein functions (Figures S1A and S1B). Importantly, the splicing pattern of Citrine-fused *SRSF1* remained similar to that of the endogenous copy, with 5 isoforms (Figure S1A), including the productively translated functional isoform 1 as well as the unproductive isoforms 2–4 (Sun et al., 2010). Like endogenous SRSF1 protein, ectopic Citrine-fused SRSF1 can downregulate total *SRSF1* RNA expression by promoting unproductive splicing to isoforms 2–4 (Figure S1B).

Having established the *SRSF1*(gDNA) and *SRSF1*(cDNA) cell lines, we next investigated quantitatively how SRSF1 modulates its own expression. In both cell lines, autoregulatory feedback on the endogenous *SRSF1* gene is expected to maintain constant SRSF1 protein levels across a modest range of ectopic expression (Figure 2A, lower panels). However, this buffering effect should saturate in the cDNA cell line once endogenous *SRSF1* is fully depleted (black curve), leading to increased total SRSF1 levels (orange curve). By contrast, for *SRSF1*(gDNA) cells, both *SRSF1* copies (ectopic and endogenous) are autoregulatory. Therefore, the total SRSF1 expression should remain the same (red curve), with a stable “ceiling” of total ectopic SRSF1 (blue curve).

Consistent with these expectations, the buffering effect from negative autoregulatory splicing could be observed in both cell lines. Inducing ectopic *SRSF1* with different concentrations of dox or the weaker affinity inducer 4-epiTC (4 epimers tetracycline, an analog of dox) produced a broad range of ectopic SRSF1 protein expression (Figures 2B and 2C). Concomitant with the increase in ectopic SRSF1 levels, we observed a decrease in endogenous SRSF1 levels in both *SRSF1*(cDNA) and *SRSF1*(gDNA) cell lines (western blot in Figures 2B and S2). We also observed a saturation of the buffering effect of endogenous copy in *SRSF1*(cDNA) cells: At ~100 ng/mL dox induction, all of the endogenous pre-mRNAs were spliced to unproductive isoforms 2–4 by 50 h (Figure S1B). These results, which are consistent with previous work done in HeLa cells (Sun et al., 2010), show that the autoregulatory feedback loop is active and can buffer variations in SRSF1 expression levels.

To obtain a more quantitative view of this behavior, we used single-cell flow cytometry to analyze SRSF1 protein levels in individual cells (Figure 2C). We induced ectopic *SRSF1* across a range of levels, fit the resulting data to a simple log-normal distribution with background (Figure S3), and extracted the mean log expression level for each condition. As expected, the Citrine-SRSF1 protein level in *SRSF1*(cDNA) cells increased monotonically with induction level (orange curve). By contrast, *SRSF1*(gDNA) cells reached a ceiling at an induction level of 300 ng/mL 4-epiTC (blue curve). The levels of ectopic and endogenous SRSF1 were quantified via gel band intensity in western blot (Figure S2). By correcting for relative protein size (487 amino acids [aa] versus 248 aa), we found the level of endogenous SRSF1 protein is ~3 times that of fully induced ectopic SRSF1 expression.

Together, these results provide a system in which autoregulated and unregulated *SRSF1* can be compared quantitatively, showing that negative autoregulatory splicing buffers SRSF1 concentrations at the steady state.

### A deep learning system allows label-free single-cell tracking in time-lapse videos

**Figure 3.**
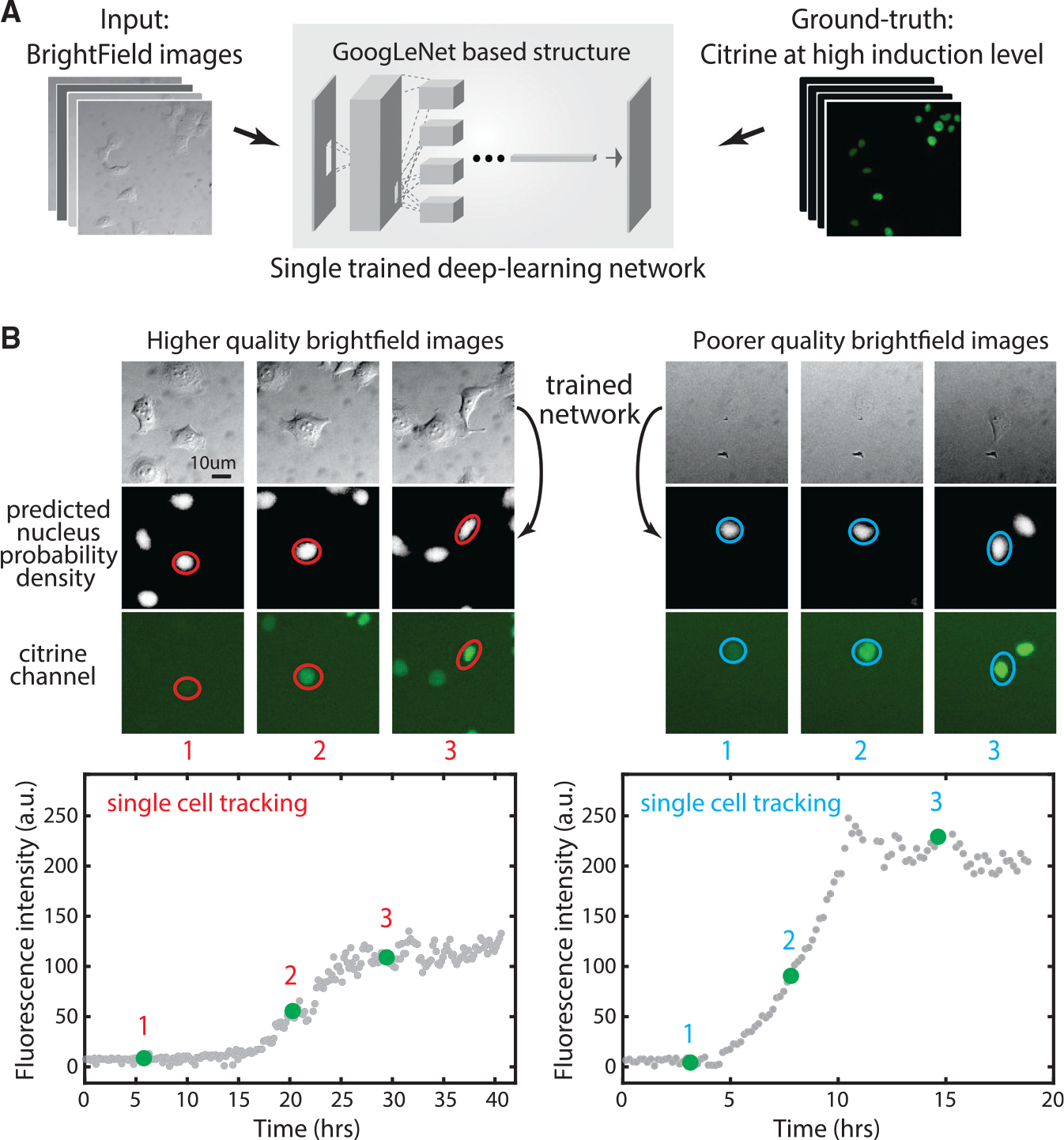
Deep-learning network enables tracking of SRSF1 accumulation in individual cells over time (A) We trained a single deep learning network using ~150 bright-field (DIC) images with their correspondent Citrine signal as the ground truth for learning nuclear location. (B) The trained network predicts nucleus images (center row) from bright-field images (top row) for 2 different example time-lapse single-cell traces (left and right panels). Note the similarity of predicted fluorescence and actual fluorescence on later images, where visible. Time points are indicated by red/blue numbers (compare with real-time tracking video curves at the bottom). Two videos show diverse bright-field background and contrast, but our trained network works on both. Red circles represent cell segmentation based on deep learning predicted nuclear probability. Left and right traces are the *SRSF1*(cDNA) cell line induced at t = 0 with 200 ng/mL 4-epiTC or 100 ng/mL dox, respectively.

We next sought to use this system to study the dynamics of negative autoregulatory splicing in individual cells in response to the induction of the ectopic constructs, in time-lapse videos. To achieve this requires identifying (segmenting) nuclei in each video frame, tracking their movements over time, and quantifying the changes in total abundance of Citrine-SRSF1 in each individual cell. Segmentation can be challenging to achieve using the Citrine fluorescence signal alone because fluorescence levels are initially too low to reliably label nuclei. To circumvent this issue, we adapted and trained the GoogLeNet deep learning system (Christiansen et al., 2018; Szegedy et al., 2015) to segment nuclei from differential interference contrast (DIC) images (Figure 3A). To train the network, we acquired ~150 paired DIC (Figure 3A, left) and fluorescence (Figure 3A, right) images of the same cells (STAR Methods), under conditions of strong nuclear Citrine expression, in which Citrine clearly labeled nuclei. After training, the network was able to segment cell nuclei from DIC images regardless of Citrine fluorescence level (Figure 3B). It also functioned across DIC images varying in brightness and contrast (Figure 3B). After segmentation, we applied a previously described cell tracking algorithm to follow individual cells over the ~40-h duration of each time-lapse video (Bintu et al., 2016; Singer et al., 2014). Finally, we extracted dynamic traces of Citrine fluorescence from each cell. Together, this deep learning-enabled protocol provides a simple and general method for label-free single-cell fluorescence tracking, avoiding the need to integrate an additional constitutively expressed fluorescent protein into the cell, minimizing cell engineering, and reducing phototoxicity during imaging.

### Negative autoregulatory splicing reduces cell-cell heterogeneity in SRSF1 levels and responsiveness

Equipped with the label-free segmentation and tracking system, we investigated the dynamics of negative autoregulatory splicing by comparing Citrine-SRSF1 dynamics in *SRSF1*(gDNA) cells to those of unregulated *SRSF1*(cDNA) cells. Specifically, we induced both cell lines at time 0 under low (200 ng/mL 4-epiTC) and high (100 ng/mL dox) induction levels, recorded DIC and fluorescence images over time, and reconstructed ~200 traces of single-cell Citrine signals for each cell line at each induction level.

**Figure 4.**
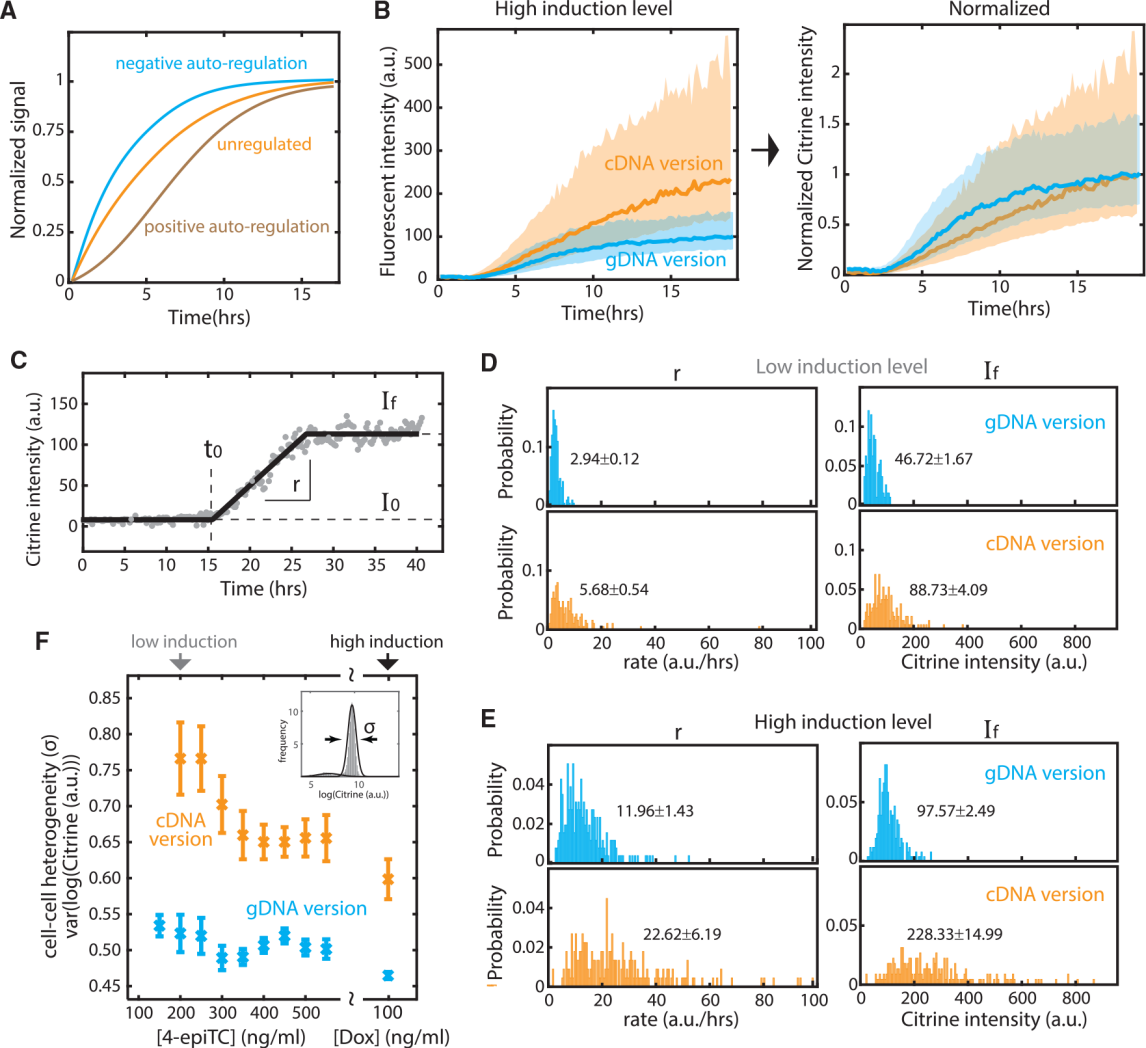
Splicing negative autoregulation reduces cell-cell heterogeneity in both the level and response rate of SRSF1 protein (A) Negative feedback accelerates response time, in comparison to unregulated or positive feedback (schematic). (B) Negative autoregulatory splicing speeds response rate and reduces cell-cell heterogeneity at high induction level (100 ng/mL dox). (Left) Solid curves are the median of 257 *SRSF1*(cDNA) and 223 *SRSF1*(gDNA) single-cell traces. The shade represents the standard deviation of the mean. (Right) The curves are normalized to final expression. (C) We fit 4 parameters to characterize time-lapse video traces. I_0_ represents background Citrine intensity and auto-fluorescence. I_f_ represents the final (steady-state) Citrine level. t_0_ denotes the time point when Citrine signal (i.e., ectopic SRSF1 level) surpasses background. r represents the response speed (slope) from I_0_ to I_f_. The example video trace is the same as in Figure 3 (left). (D) Distributions of rate and final intensity for 191 gDNA and 188 cDNA traces with 200 ng/mL 4-epiTC added at t_0_ (see I_0_ and t_0_ distribution in Figure S4). (E) Similar distributions for gDNA and cDNA traces with 100 ng/mL dox added at t_0_ (see I_0_ and t_0_ distribution in Figure S4). The labeled text denotes the median and the standard error of the mean. At both induction levels, the autoregulatory (gDNA) system exhibits a tighter distribution of final equilibrium SRSF1 levels and response rates. The negative splicing feedback loop thus reduces cell-cell heterogeneity both in final level and dynamics. (F) Flow cytometry data confirms that the autoregulatory (gDNA) system exhibits lower cell-cell heterogeneity across a wide range of induction levels. As in Figure 2C, we induced gDNA and cDNA cells for >24 h, fit the Citrine intensity with a Gaussian curve (Figure S3), and used the standard deviation parameter from the Gaussian fit to represent the variance of ectopic SRSF1 level between cells. Error bars represent the standard error of the mean from 9 experimental replicates.

Negative autoregulation at the transcriptional level was previously shown to accelerate response times and to reduce cell-cell heterogeneity (illustrated in Figure 4A) (Alon, 2006; Rosenfeld et al., 2002). Time-lapse videos revealed that negative autoregulatory splicing has a similar impact: *SRSF1*(gDNA) cells reached steady-state levels faster than *SRSF1*(cDNA) cells (Figure 4B).

Furthermore, these traces (Figures 4B and S4A) also showed that negative autoregulatory splicing reduces cell-cell heterogeneity. *SRSF1*(gDNA) cells exhibited a tighter distribution of temporal traces compared to *SRSF1*(cDNA) cells. To quantitatively examine cell-cell heterogeneity at different induction levels, we compared *SRSF1*(gDNA) and *SRSF1*(cDNA) curves with the distribution of four parameters extracted from each video trace (Figure 4C): I_0_ and I_f_, characterizing the initial and final fluorescence levels respectively; t_0_, the time at which the rising signal occurred; and r, the slope of the rise. At low induction levels (Figure 4D), the standard deviation of r and I_f_ relative to their median values increased from 0.04 to 0.036 in *SRSF1*(gDNA) cells to 0.10 and 0.046 in *SRSF1*(cDNA) cells, respectively. Similarly, at high induction levels (Figure 4E), variability in r and I_f_ increased from 0.12 to 0.026 in *SRSF1*(gDNA) cells to 0.27 and 0.066 in *SRSF1*(cDNA) cells. We also analyzed a broader range of 4-epiTC concentrations by flow cytometry (Figure S3), and confirmed that negative autoregulatory splicing reduces cell-cell heterogeneity of I_f_ across a wide range of induction levels (Figure 4F).

The delay before activation, t_0_, varied systematically with the induction level, decreasing from ~13 h at low induction levels to ~3 h at the highest induction levels (Figures S4B and S4C). Three hours is comparable to the total expected time required to synthesize mature SRSF1 protein (Milo et al., 2010). The longer 13-h delay may reflect the bursty nature of transcription, which can produce extended intervals between transcriptional bursts (Larsson et al., 2019; Raj et al., 2006), and variable 4-epiTC/dox induction strength and absorption efficiency between cells. This could also explain why we did not observe an accelerated response time for the *SRSF1*(gDNA) circuit at low induction levels (compare Figures S4A and 4B). Notably, t_0_ did not differ between the *SRSF1*(gDNA) and *SRSF1*(cDNA) cell lines (Figures S4B and S4C), suggesting that it is controlled by factors independent of the splicing regulatory circuit. Similarly, the heterogeneity and level of background signal I_0_ remained the same for *SRSF1*(gDNA) and *SRSF1*(cDNA) cells regardless of induction level (Figures S4B and S4C), suggesting that the sum of autofluorescence and promoter leakage was similar between the two circuits.

Taken together, these results demonstrate that negative autoregulatory splicing can speed response rates and reduce cell-cell heterogeneity in response rate and expression level. These effects are similar to those of other well-known negative autoregulatory feedback loops (Becskei and Serrano, 2000; Rosenfeld et al., 2002, 2007). Despite the similarity, negative autoregulatory splicing has a feature potentially distinct from other types of negative feedback—its ability to simultaneously affect a large and variable load of target pre-mRNAs (Figure 1B).

### Negative autoregulatory splicing modulates response of *SRSF1* level to splicing load

**Figure 5.**
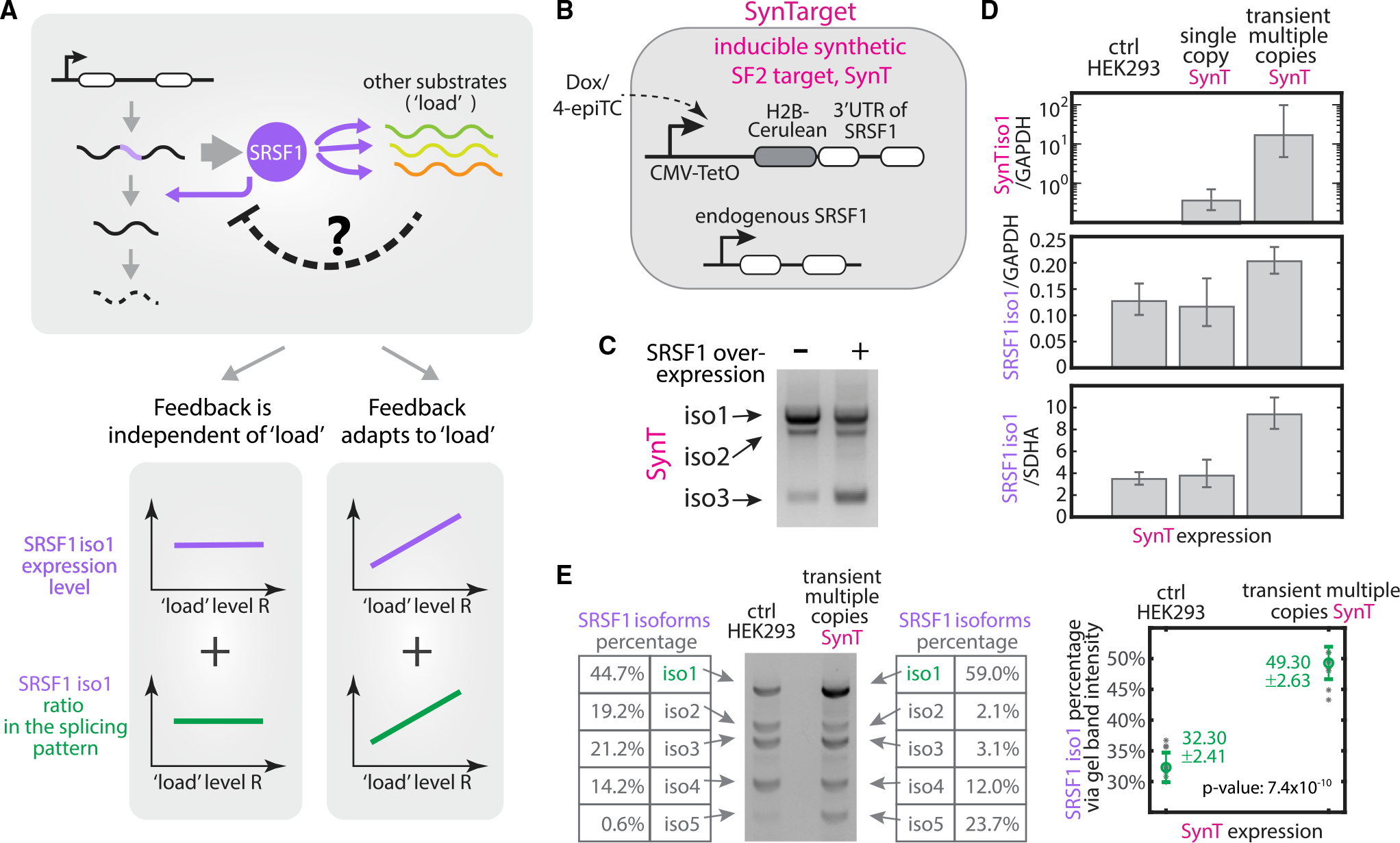
Splicing negative autoregulation modulates its feedback strength in response to variable total substrate load (A) Two possible outcomes in response to total substrate load. (Left) Robust feedback scheme: The total *SRSF1* level (purple line) and its splicing pattern (green line) remain constant across a broad range of substrate levels. The amount of SRSF1 involved in negative feedback is independent of load level. (Right) Adaptive feedback scheme: more SRSF1 is produced (purple and green curves) via weakening negative autoregulatory splicing (i.e., dashed negative arrow in the top gray box), as increased substrate load titrates away available SRSF1 in the cell. (B) The inducible synthetic SRSF1 target (SynTarget [*SynT*]) cell line contains H2B-Cerulean fused with the spliceable 3′ UTR of SRSF1. This synthetic gene is expressed under a Tet-On CMV promoter and stably integrated at the Flp-In locus in a T-REx HEK293 cell line. (C) *SynT* is a splicing target of SRSF1. We used RT-PCR and gel-imaged 3 isoforms of *SynT* cells with 100 ng/mL dox (left lane) and of *SynT* cells with transiently transfected *SRSF1*(cDNA) plasmid in 100 ng/mL dox (right lane). We found that SRSF1 overexpression promotes the splicing of SynT, increasing the expression of short isoforms. (D) We induced *SynT* at different levels and quantified the concentration of *SynT* isoform 1 (top row) and the functional *SRSF1* isoform 1 (bottom 2 rows) by qRT-PCR (see qPCR qualification in Figure S6). We found that *SRSF1* levels remained unchanged by expression from a single copy of *SynT* (center column, by inducing the stably integrated *SynT* with 100 ng/mL dox), but increased ~50% when multiple *SynT* copies were induced in the same cell (right column, by transiently transfecting *SynT* plasmid with 100 ng/mL dox). qPCR results were verified by normalizing to 2 housekeeping genes, *GAPDH* and *SDHA*, respectively. The data represent the exponential logarithmic mean of normalized qPCR reads. Error bars represent the minimum and maximum values over 3–10 experimental replicates. (E) *SRSF1* splice isoform pattern changes in response to increased total substrates. We quantified *SRSF1* isoforms using RT-PCR and analyzed the gel band intensity by Bio-Rad ChemiDoc Image Lab 6.0 band analyzer. Two gel band examples are presented—one from HEK293 control (left), the other from transient multiple copies of *SynT* (right). Multiple copies of *SynT* trigger ~50% more *SRSF1* isoform 1 (i.e., functional unspliced isoform) through splicing. The data represent the median of gel band intensity percentage reads and error bars represent the standard deviation over 6–7 experimental replicates.

In principle, *SRSF1* negative autoregulatory splicing could operate in two opposite regimes that differ in their response to increased substrate (pre-mRNA) load (Figure 5A). In “robust” mode, the feedback strength would be independent of the load level. Despite the competition between distinct pre-mRNA substrates, SRSF1 protein is abundant in the cell, and SRSF1 production maintains a constant concentration in the cell. Alternatively, in an “adaptive” mode, the feedback loop would modulate its negative autoregulatory strength, tuning the SRSF1 level in response to total substrate load. In this case, increased substrate load, like a sponge, would dilute the available SRSF1 protein per substrate molecule, reducing the feedback strength, generating more functional *SRSF1* isoform 1, and thereby producing more SRSF1 protein.

To discriminate between these two regimes and investigate whether and how negative autoregulatory splicing responds to perturbations of load, we introduced a synthetic target (*SynT*) of SRSF1 in HEK293 cells (Figure 5B) under a dox-inducible CMV promoter. *SynT* contains the spliceable 3′ UTR of *SRSF1*, fused with a fluorescent protein, H2B-cerulean, at the 5′ end. It generates three isoforms (Figure S5), with the same splicing junction as *SRSF1* isoforms (Figures 1A and S1A). The overexpression of SRSF1 protein (through transiently transfecting the cDNA version of *SRSF1*, as in Figure S1B) altered the splicing pattern of *SynT* toward more spliced isoforms (Figure 5C), consistent with *SynT* acting as a SRSF1 target.

To determine the response of *SRSF1* splicing to various *SynT* loads, we need to quantify the splicing pattern of *SRSF1*, as well as the expression level of the only functional isoform of *SRSF1*, isoform 1, across different *SynT* induction levels (Figure 5A, bottom). The splicing pattern can be quantified using RT-PCR (Figures 5C and S5) by amplifying all of the isoforms at once with a single primer set targeting the 5′ and-3′ end of the gene (STAR Methods). The amount of *SRSF1* isoform 1 can be quantified using qRT-PCR by a primer set (Figure S1A) specifically targeting isoform 1 without amplifying isoforms 2–5 (Figure S6A). The quantitative calibration of the designed qPCR primers is verified in the dilution curve shown in Figure S6B.

We then tested the response of *SRSF1* splicing to two different ectopic expression levels of *SynT*: A site-specifically integrated single copy of *SynT* (Figure 5D, single-copy *SynT*), and transiently transfected multiple copies of the *SynT* that together produce ~20–100 times more *SynT* Figure 5D, transient multi-copy *SynT*). The latter ensures that the ectopic *SynT* load effectively competes with the large number of endogenous SRSF1 target substrates (Das and Krainer, 2014). As shown in Figure 5D (middle and lower panels), we observed no significant change in *SRSF1* isoform 1 levels in response to the single-copy perturbation. By contrast, *SRSF1* isoform 1 levels increased ~2-fold in response to transient, multi-copy *SynT* transfection.

Negative autoregulatory splicing contributed to this increased *SRSF1* isoform 1 level. As shown in Figure 5E, when the *SynT* level increased, a higher fraction of *SRSF1* pre-mRNA remained unspliced, producing more functional isoform 1. Based on gel band intensity quantification, the ratio of isoform 1 increased approximately 1.53 times, consistent with the readout from Agilent Bioanalyzer 2100 (Figure S7). This result suggests that the overexpression of SRSF1 substrates can reduce SRSF1 availability, impeding the unproductive self-splicing of *SRSF1*, and thereby increasing the level of functional SRSF1 isoform 1. Notice that the isoform 1 ratio increment (Figure 5E) is smaller than the increased *SRSF1* isoform 1 level (Figure 5D), indicating the existence of other regulation layers on *SRSF1* production, apart from the negative autoregulatory splicing (see more in the Discussion section).

**Figure 6.**
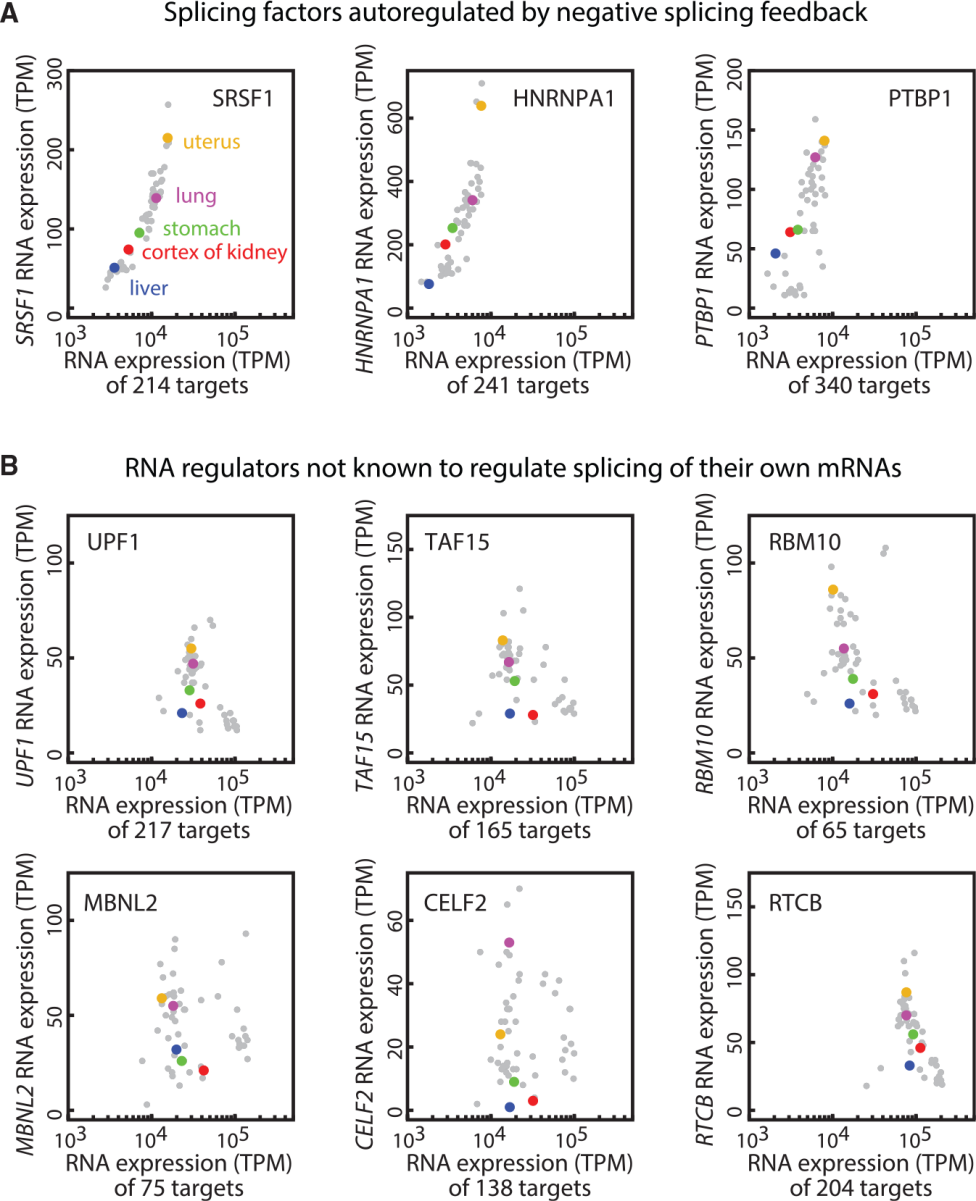
The adaptation function of splicing negative autoregulation responds to variations in endogenous targets load (A) The expression level of splicing factors positively correlates with their target expression across 53 human tissue types (gray dots), in which 5 example tissues (uterus, lung, cortex of kidney, stomach, liver) are labeled in distinct colors. The splicing factor RNA expression levels (TPM) were extracted from the GTEx database. The respective target genes are selected based on POSTAR2, specifically, the top 2% “binding site records” in each CLIP database (STAR Methods). The 3 splicing factors SRSF1, hnRNPA1, and PTBP1 are autoregulated via negative splicing feedback. (B) The RNA regulation proteins not known to regulate splicing of their own mRNAs do not show correlative patterns as in (A).

*SynT* is a synthetic target. We further checked whether *SRSF1* splicing similarly responds to variations in the load presented by its endogenous targets. Specifically, we obtained a list of SRSF1 target genes from the cross-linking and immunoprecipitation with high-throughput sequencing (CLIP-seq) database POSTAR2 (Zhu et al., 2019), and the corresponding transcript levels (transcripts per million, TPM) of both *SRSF1* and its CLIP-based target genes from GTEx (www.gtexportal.org) across 53 human tissue types. In addition to *SRSF1*, we included two other splicing factors, *hnRNPA1* and *PTBP1*, with conserved autoregulatory negative splicing feedback loops (Ni et al., 2007; Wollerton et al., 2004). In contrast, we picked six RNA binding proteins not known to regulate splicing of their own mRNAs: UPF1, RNA helicase, and ATPase (involved in mRNA nuclear export and surveillance); TAF15, TATA-binding protein-associated factor (involved in RNA polymerase II transcription); RBM10, RNA-binding motif (involved in splicing regulation); RTCB, RNA ligase; MBNL2, RNA splicing regulator; and CELF2, RNA splicing regulator. The abundance of the three negatively autoregulated splicing factors positively correlated with that of their targets (Figures 6A, S8A, and S9A), while the six other RNA regulators did not present any clear correlation (Figures 6B, S8B, and S9B). This lack of correlation occurred across multiple CLIP-seq data background-extraction methods (STAR Methods). The quantitative correlation coefficients are listed in Figure S11. Note that these data do not rule out roles for other potential regulatory interactions that could also contribute to these correlations (see Discussion).

Taken together, these results indicate that substrate load can modulate negative autoregulatory splicing feedback. This load-adaptive feedback scheme helps ultra-conserved splicing factors to adapt their own protein expression levels to variable substrate levels across diverse tissues (Hanamura et al., 1998; Lareau et al., 2007; Zahler et al., 1992).

### A mathematical model explains the dynamics and function of negative autoregulatory splicing

Having shown experimentally that negative autoregulatory splicing accelerates SRSF1 response times and enables their adaptation to substrate load, we sought to understand how these features arise from the autoregulatory architecture. We developed a mathematical model describing the dynamics of SRSF1 with and without feedback, and fit the model to SRSF1 dynamics observed in time-lapse videos of the *SRSF1*(gDNA) and *SRSF1*(cDNA) cell lines.

**Figure 7.**
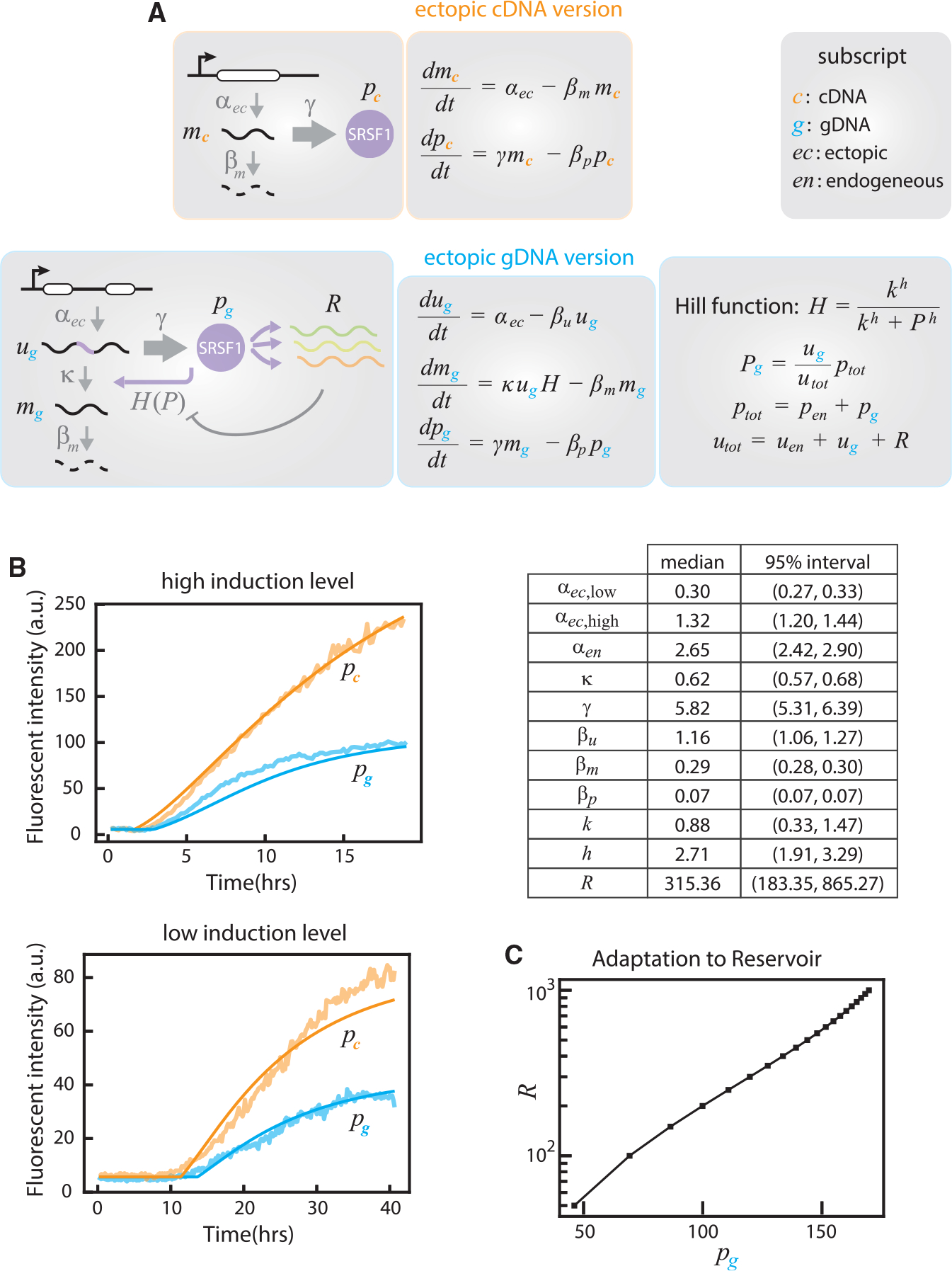
A minimal mathematical model describes splicing regulation with and without negative feedback (A) Differential equation sets for the regulation of ectopic *SRSF1*(cDNA) (without feedback) and *SRSF1*(gDNA) (with feedback) cell lines. Unspliced pre-mRNA, functional mRNA, and SRSF1 protein are labeled *u*, *m*, and *p*, respectively. Subscripts are defined at left. As each ectopic copy is stably integrated into the same genomic locus of HEK293 cells, they share the same transcription rate (*α*), translation rate (*γ*), and degradation rate (*β*) between cell lines. Note that gDNA system incorporates a Hill function *H(P)* to represent the dependence of splicing activity on SRSF1 protein. For details, see STAR Methods. (B) Fits of median time-lapse traces from Figures 4B and S4A to the equations in (A). Fitted curves are shown with smooth solid lines. Bestfit parameter estimates are shown in the table and Figure S12. (C) Negative splicing autoregulation adapts to load. Using the fitted parameters, the model predicts a positive correlation between target load and SRSF1 level, *p*, similar to experimental observations (Figure 5).

We formulated differential equations describing the dynamics of unspliced pre-mRNA *u*, functional mRNA isoform *m*, and protein *p* from the endogenous (subscript *en*) and ectopic (subscript *ec*) copies (Figure 7A). For both sets of equations, we assumed the same constant production rate (α) for either of the two ectopic constructs, controlling production of either *m*_*ec*_ for *SRSF1*(cDNA) or *u*_*ec*_ for *SRSF1*(gDNA). We also assumed that the translation of *p* is linearly related to the corresponding mRNA concentration *m*, at rate g, and that all of the species undergo first-order degradation with rate constant β.

We define two sets of equations, with and without feedback. In the case of *SRSF1*(cDNA), the functional isoform *m*_*ec*_ is not subject to negative autoregulatory splicing. Since the endogenous components do not have any regulatory effect, modeling the dynamics of *m*_*ec*_ and *p*_*ec*_ is sufficient (Figure 7A, ectopic cDNA version; STAR Methods). In contrast, the *SRSF1*(gDNA) cell line features negative feedback affecting the splicing of *u*_*ec*_ to *m*_*ec*_ via both the endogenous and ectopic SRSF1 proteins (Figure 7A, ectopic gDNA version and endogenous copy; STAR Methods). Since SRSF1 also acts on many other target genes, collectively denoted *R*, we reasoned that the level of SRSF1 available for autoregulatory splicing would be reduced by competition with *R*. Therefore, we modeled negative feedback by a Hill function *H(p)*, where the effective protein level acting on *SRSF1* transcripts depends on the abundance of those transcripts relative to those of the reservoir. The resulting differential equations are summarized in Figure 7A; more details on the derivation are given in STAR Methods.

We then fit the model parameters to the averaged traces in Figure 4B using a Bayesian inference framework (Figure 7B; STAR Methods). The data comprise ectopic SRSF1 protein levels for the *SRSF1*(cDNA) and *SRSF1*(gDNA) lines at both low and high induction levels (Figures 4B and S4). Shared biochemical parameters (e.g., translation and degradation rates) are fit jointly across all of the conditions. Parameters specific to the negative feedback case are *κ*, the efficiency of splicing to the unproductive isoform; *k*, the substrate concentration that produces half-maximum splicing activity; *h*, the Hill coefficient; and *R*, the reservoir level (STAR Methods). Only the ectopic production rate was allowed to change between low and high induction levels.

We computed probability distributions for each parameter (Figure S12) and identified median values and confidence intervals (Figure 7B, table). These values were approximately consistent with independent parameter estimates. In particular, the ratio between production rates of the ectopic and endogenous *SRSF1* copy in *SRSF1*(gDNA) cells (i.e., α_*ec*_ versus α_*en*_) matched that obtained from the western blot in Figure S2. Similarly, the fit degradation rate values generate half-lives of approximately 35 min for *u*, 2.4 h for *m*, and 10 h for *p*, broadly consistent with values in other studies (Milo and Phillips, 2015; Moulton et al., 2014). Finally, the fitted value of *k* suggests that 62% of transcripts are spliced to isoform 1, generally consistent with the percentage seen in multi-copy *SynT* transfection (Figure 5E).

The fitted parameters can provide deeper insight into the biology of SRSF1 regulation. The Hill coefficient value *h* ~ 2–3 suggests ultra-sensitivity of SRSF1 activity. The estimated reservoir level *R* of nearly 300 suggests approximately several hundred additional SRSF1 target transcripts (with relatively high abundances) per *SRSF1* transcript in this cell type, also consistent with typical values in a variety of cell types (Figures 6A, S8A, and S9A). Finally, using the model to compute the steady-state ectopic protein level as a function of *R* reveals that SRSF1 level adapts to target load (Figure 7C), consistent with experimental results (Figures 6A, S8A, and S9A).

## DISCUSSION

Negative autoregulation is a prominent feature of many splicing regulatory systems. To understand its functional impact, we constructed three HEK293 cell lines, with inducible gDNA (autoregulated), cDNA (unregulated), and *SynT* (synthetic SRSF1 targets) and quantitatively investigated the dynamic function of negative autoregulatory *SRSF1* splicing in individual cells. By combining deep learning-based single-cell video analysis (Figure 3) with flow cytometry, qRT-PCR, and RT-PCR, we found that negative autoregulatory splicing can stabilize SRSF1 levels independent from its own transcription strength, reduce cell-cell heterogeneity in both SRSF1 levels and response times (Figure 4), and adapt to changes in target load (Figures 5, S8, and S9). Although we focused on SRSF1, the approach presented here can be extended to study other splicing factors.

A particularly interesting aspect of negative splicing autoregulation, compared to better-known negative autoregulatory transcriptional feedback, is its ability to homeostatically control splicing activity in response to changes in total substrate load. In contrast to a transcriptional circuit facing a relatively fixed number of binding sites, splicing factors operate on variable amounts of substrate, including their own pre-mRNAs as well as other target genes (Figure 1B). Thus, to maintain a constant splicing activity, negative autoregulatory splicing buffers SRSF1 effective concentration against variations in both *SRSF1* pre-mRNAs level (Figures 2 and 4) as well as their target levels (Figures 5 and 6). Noticeably, even though both *SRSF1* and their target *SynT* overexpression increases absolute SRSF1 levels, they produce distinct and opposite effects on *SRSF1* splicing patterns (Figures S1B and S5D), with more spliced *SRSF1* isoforms produced when *SRSF1*(cDNA) was overexpressed (Figure S1B) and more unspliced *SRSF1* isoforms when *SynT* was overexpressed (Figure 5E). These counterintuitive results confirm that the cell maintains subsaturating SRSF1 levels.

In addition to the experimental data, mathematical modeling provided further insights into SRSF1 autoregulation. First, it shows that experimentally observed effects can arise from basic aspects of splicing and transcriptional regulation more generally, by phenomenologically incorporating Hill functions into the differential equations listed in Figure 7A to describe the concentration dynamics of involved RNA (spliced, unspliced) and protein molecules. Second, the model provides a unified understanding of the sometimes counterintuitive responses of *SRSF1* to different perturbations driven by autogenous splicing and feedback (i.e., *SRSF1* overexpression in Figure 7B, versus perturbations in other target substrates, the amount of reservoir in Figure 7C). Finally, the model identified biochemical parameters, such as the half-lives of SRSF1 protein and its spliced/unspliced RNAs, the co-transcriptional splicing efficiency, as well as the amount of target substrates, that recapitulate experimental observations and can help to inform a more quantitative understanding of splicing dynamics.

Negative autoregulatory splicing is not the only factor affecting SRSF1 expression. Our experimental data and mathematical modeling do not rule out more complex mechanisms. For instance, the ratio of isoforms 2–5 in *SRSF1* splicing pattern varies when we perturb the SRSF1 level (Figure S1B) or SRSF1 substrate load (Figure 5E), indicating that SRSF1 concentration (either overexpression or depletion) is not the only factor affecting *SRSF1* splicing. In addition, a previous study (Sun et al., 2010) revealed translational level regulation on SRSF1 expression. Further molecular analysis will be necessary to fully characterize mechanistic aspects of autoregulation.

This analysis does not explicitly incorporate the non-uniform spatial distribution of SRSF1 in the nucleus. SRSF1 concentrates in interchromatin granule clusters called speckles (Lamond and Spector, 2003; Misteli et al., 1997) that exhibit dynamic structures (Misteli et al., 1997). Recent work suggests that the spatial distribution of splicing factors correlates with active transcription hubs in the nucleus (Ding and Elowitz, 2019; Quinodoz et al., 2018). Because most splicing occurs co-transcriptionally (Bentley, 2014; Das et al., 2007; Rosonina and Blencowe, 2002), negative autoregulatory splicing should, in principle, feedback on the local SRSF1 concentration within a subnuclear neighborhood, rather than the global concentration averaged over the nucleus as a whole. It thus remains unclear how splicing factors balance their local and global concentrations in the nucleus. In the future, it will be interesting to develop a more complete analysis of negative autoregulatory splicing that includes SRSF1 subcellular spatial distribution and may provide an integrated view of how cells maintain constant effective SRSF1 concentrations despite heterogeneity in their subnuclear spatial distributions.

More generally, negative autoregulatory splicing has the potential to affect diverse cellular functions. For instance, splice regulators could impact cell physiology by regulating “poison” exons, among other mechanisms (Thomas et al., 2020; Leclair et al., 2020). Splice regulation could also be useful in synthetic circuits in which microRNAs are expressed from introns (Strovas et al., 2014). These natural and synthetic systems both suggest the importance of further investigation into the actual and potential roles of splicing feedback systems.

### Limitations of the study

Splicing is a complicated process. Both our experiments and mathematical models focus only on the negative autoregulatory aspect of splicing (with transcriptional control). They do not analyze the roles for other regulatory mechanisms, such as NMD regulation, translational regulation, and epigenetic regulation. A complete understanding of splice regulation will eventually need to account for multiple regulatory systems. In addition, we focused here on quantifying the dynamics of SRSF1 autoregulation in the HEK293 cell line. Cell lines and cell types differ in the expression of splicing components, and autoregulatory splicing dynamics could correspondingly vary between different cell types or in more complex contexts, such as specific tissues.

## STAR★METHODS

### RESOURCE AVAILABILITY

#### Lead contact

Further information and requests regarding resources and reagents can be directed to Michael Elowitz (melowitz@caltech.edu).

#### Materials availability

Cell lines generated in this study are available from the lead contact upon request.

#### Data and code availability

- Data generated in this study are available from the lead contact upon request.
- All data reported in this paper will be shared by the lead contact upon request.
- Original codes are deposited and publicly accessible. DOI is listed in the Key resources table.
- Any additional information required to reanalyze the data reported in this paper is available from the lead contact upon request

### EXPERIMENTAL MODEL AND SUBJECT DETAILS

#### Plasmids

*SRSF1(gDNA)*, or *SRSF1*(cDNA), or *SynT* was cloned into a Flp-In^™^ pFRT expression vector (Life Technologies) under an inducible CMV-TO promoter. *SRSF1(gDNA)* incorporated the same sequence as the endogenous *SRSF1* gene (based on gDNA sequencing), including the 3’UTR part. *SRSF1(cDNA)* incorporated the sequence of mRNA of endogenous *SRSF1* (without the 3’UTR site) with the BGH polyadenylation sequence in the pFRT vector. *SynT* contained H2B-Citrine fused with the 3’UTR site of the endogenous *SRSF1* gene.

#### Cell lines

Flp-In^™^ T-Rex^™^ HEK293 cells (Life Technologies, we did not test for mycoplasm) were cultured following the manufacturer’s protocol. For transfection, the cells were pre-plated in 24-well plates with 80% confluency. We added 800–1000 ng of plasmid (*SRSF1*(gDNA) or *SRSF1*(cDNA) or *SynT*) using the Lipofectamine LTX plasmid transfection reagent (ThermoFisher Scientific) and changed the culture media to Opti-MEM^™^ Reduced Serum Medium (ThermoFisher Scientific). Cells were left in the incubator overnight, then trypsinized (using 0.25% Trypsin-EDTA (Thermo Fisher Scientific)) into new 6-well plates with complete culture media the next day. These cells were then cultured for 1–2 weeks with 100 ug/ml Hygromycin. The surviving transfected cells were subcloned by limiting dilution.

### METHODS DETAILS

#### Time-lapse microscopy imaging and data analysis

24-well 10 mm diameter glass No. 1.5 coverslip plates (MatTek Corp.) were coated with 5 ug/ml Human Fibronectin (Oxford Biomedical Research, Rochester Hills, MI) in PBS buffer for 1hr at room temperature. Fibronectin was then aspirated, and 4,000 – 10,000 cells were plated in the coated 24-well plate with complete cell media. The plate was manually swayed (Hui and Bhatia, 2007) to uniformly spread the cells, and left in the incubator for 2–3 h before imaging. Details of microscopy have been described previously (Nandagopal et al., 2019). For each movie, 40–60 stage positions were picked manually, and YFP and differential interference contrast (DIC) images were acquired every 10 min with an Olympus 20x objective using automated acquisition software (Metamorph, Molecular Devices, San Jose, CA). The details of cell tracking algorithm was in (Bintu et al., 2016; Singer et al., 2014).

#### Flow cytometry

Experimental procedures and data analysis for flow cytometry have been described previously (Nandagopal et al., 2019).

#### Transient transfection

Cells were plated at 50% confluency in 24-well plates and grown to 80% confluency overnight. We added 800–1000 ng of plasmid (*SRSF1*(gDNA) or *SRSF1*(cDNA) or *SynT*) using the Lipofectamine LTX plasmid transfection reagent (ThermoFisher Scientific) and changed the culture media to Opti-MEM^™^ Reduced Serum Medium (ThermoFisher Scientific). Cells were left in the incubator for 5.5 h, then trypsinized (by 0.25% Trypsin-EDTA (Thermo Fisher Scientific)) off the plates and resuspended in PBS (Phosphate-Buffered Saline buffer, Thermo Fisher Scientific). These cells were centrifuged, and the pellet washed 3x with PBS buffer to remove Trypsin. The final cell pellets were either dissolved for RNA extraction, or frozen and stored at −80C.

#### RT-PCR

We extracted total RNA using the RNeasy Mini Kit (Qiagen), and used 500 ng - 1 ug RNA to make cDNA using the iScript cDNA Synthesis Kit (Bio-Rad Laboratories, Hercules, CA). 0.1 of cDNA (i.e. 1ul after diluting cDNA 10x) was used in the PCR reaction, using AccuPrime^™^ Pfx SuperMix (Thermo Fisher Scientific) with an annealing temperature at 62.5 degree for 35 cycles. PCR primers are listed in Table S1. 3–8 ul of the PCR product was then run on a 1–2% Agarose gel. Gel band intensity was analyzed by Bio-Rad ChemiDoc Image Lab 6.0 band analyzer.

#### PT-qPCR

RNA was extracted as in RT-PCR. We then used 1 ug RNA to make cDNA using the SuperScript^™^ III First-Strand Synthesis kit (Thermo Fisher Scientific) with all gene-specific primers (*SRSF1*, *SynT, GAPDH*, and *SDHA* isoform1 gene-specific primers). We used gene-specific cDNA for qPCR, rather than random cDNA, to minimize the influence between isoforms with similar sequences. Experimental procedures and data analysis of qPCR were performed as described previously (Nandagopal et al., 2019). All primers are listed in Table S1.

#### Western blot

Frozen or fresh cell pellets (with 10^6^ cells) were denatured using 200ul SDS loading buffer (1x sodium dodecyl sulfate (Sigma-Aldrich), 1x protease inhibitor, 4 mM EDTA) and heated for 5mins at 68°C in a water bath. The heated cells were then centrifuged at 55,000 rpm for 1hr at 4°C. 30 ul of the supernatant was loaded onto a NuPAGE^™^ 4–12% Bis-Tris Protein Gel (Thermo Fisher Scientific) and transferred using iBlot^™^ Transfer Stack to nitrocellulose (Thermo Fisher Scientific) following the manufacturer’s protocol. The blot was then blocked in 1xTBST, 5% dry milk, 2% BSA for 1 h at room temperature, followed by overnight incubation at 4°C with the primary antibody, anti-SRSF1 antibody (ab133689) at 1:1000 and anti-GFP (ab1218) at 1:2000, together. The next day, the blot was washed with 1x TBST three times, then incubated with anti-rabbit and anti-mouse HRP conjugated secondary antibody (GE Healthcare Life Sciences) at 1:2000 for 2hrs at 4°C. The blot was then washed with 1x TBST at room temperature for 1hr and five more times for 3 min. Gel bands were detected using SuperSignal West Femto Chemiluminescent Substrate (Thermo Fisher Scientific) and analyzed using a Bio-Rad ChemiDoc Image Lab 6.0 band analyzer.

### QUANTIFICATION AND STATISTICAL ANALYSIS

#### CLIP-seq data analysis

CLIP-seq database is downloaded from POSTAR2 (Zhu et al., 2019). The TPM data of target gene expression across 53 human tissue types is downloaded from GTEx (www.gtexportal.org).

As shown in Figures S10A and S10B, CLIP-seq has found more than 10,000 targets for most RNPs. With such a big number, when we add up the TPM of all the targets, the sum is directly comparable to total transcriptome (Figures S10C and S10D). To minimize the effect of targets with weak and non-specific binding, we only pick the strongest ones based on “Binding site records” in the CLIP-seq database.

We define two different ways to set up the threshold for binding-strength selection, based on either ‘percentile’ or ‘absolute’ modes. In the ‘percentile’ mode, for each RNP, we pick the target genes with its top 2% (Figure 6) or 5% (Figure S8) “Binding site records”. By changing from 2% to 5%, the number of selected CLIP-seq target genes is doubled. In the ‘absolute’ mode (Figure S9), for each RNP, we pick the threshold of “Binding site records” that can achieve to set the median TPM of selected target genes in between 2% and 5% of total transcriptome expression. In short, ‘percentile’ mode depends on the “Binding site records” distribution of each RNP, while ‘absolute’ mode aims for a comparable TPM sum of selected targets between RNPs.

As shown in Figures S8 and S9, despite the number of selected targets are different across various threshold setting (for instance, *hnRNPA1* changed from 241, to 603, then 2049), the data pattern (indicating the adaption function of negative autoregulatory splicing) persists.

This pattern starts to diminish with a higher threshold, where the TPM sum of selected targets is beyond 10% of total transcriptome.

#### Mathematical model with and without negative splicing feedback

For the cDNA construct, ectopic mRNA transcripts *m*_*ec*_ are generated with some production rate α_*ec*_, while protein *p*_*ec*_ is translated from mRNA with rate γ. Both species are degraded by first-order kinetics with rate constants *β*_*m*_ and *β*_*p*_ respectively. Therefore, their dynamics are described by the following set of differential equations:

$$\frac{\text{d}m_{ec}}{\text{d}t}={\text{α}}_{\text{ec}}-\beta_{m}m_{ec}$$

$$\frac{dp_{ec}}{dt}=\text{γ}m-\beta_{p}p_{ec}$$

For the gDNA construct with negative autoregulation of splicing, we must consider not only mRNA and protein levels *m* and *p* but also unspliced pre-mRNA levels *u*. Here, ectopic *u*_*ec*_ is generated with production α_*ec*_ and endogenous transcripts *u*_*en*_ with rate α_*en*_. These transcripts are spliced productively to *m*_*ec*_ and *m*_*en*_ with rate *k*. This splicing is regulated by negative feedback from the effective SRSF1 protein level *P*, which we describe phenomenologically by a Hill function

$$\frac{k^{h}}{k^{h}+P^{h}}$$

The total protein level comprises both endogenous and ectopic SRSF1, or *p*_tot_ = *p*_en_ + *p*_ec_. However, SRSF1 acts not only on ectopic and endogenous *SRSF1* transcripts but also on other target RNAs, or a “reservoir” *R*. Therefore, the effective protein level for any given target is not the total amount. SRSF1 not only regulates the ectopic and endogenous *SRSF1* transcripts but also acts on other target RNAs, or a “reservoir” *R*. We assume that the effective protein level for a given target is determined by the relative proportion of the target RNA. Defining the total level of unspliced targets by *u*_tot_ = *u*_en_ + *u*_ec_, the effective protein level acting on endogenous *SRSF1* is

$$P_{\text{en}}=\frac{u_{\text{en}}}{u_{\text{tot}}}p_{\text{tot}}$$

and similar for ectopic transcripts. Pre-mRNAs undergo first-order decay with rate constant β_u_; other biochemical parameters - translation rate as well as mRNA and protein degradation rates - are shared with the no-feedback model. The dynamics are given by the following set of differential equations:

$$\frac{\text{d}u_{ec}}{\text{d}t}={\text{α}}_{\text{ec}}-\beta_{\text{u}}u_{\text{ec}}$$

$$\frac{\text{d}m_{ec}}{\;\text{d}t}=ku_{ec}\cdot \frac{k^{h}}{k^{h}+{\left(\frac{u_{\text{ec}}}{u_{\text{ec}}+u_{\text{en}}+R}\left(p_{\text{ec}}+p_{\text{en}}\right)\right)}^{h}}$$

$$\frac{dp_{\text{ec}}}{\text{dt}}=\text{γ}m_{\text{ec}}-\beta_{u}p_{\text{ec}}$$

The equations for the endogenous species are analogously defined:

$$\frac{\text{d}u_{\text{e}n}}{\;\text{d}t}={\text{α}}_{\text{en}}-\beta_{\text{u}}u_{\text{en}}$$

$$\frac{\text{d}m_{en}}{\;\text{d}t}=ku_{en}\cdot \frac{k^{h}}{k^{h}+{\left(\frac{u_{\text{en}}}{u_{\text{ec}}+u_{\text{en}}+R}\left(p_{\text{ec}}+p_{\text{en}}\right)\right)}^{h}}-\beta_{m}m_{en}$$

$$\frac{dp_{\text{en}}}{\text{dt}}=\text{γ}m_{en}-\beta_{p}p_{\text{en}}$$

#### Model parameters

We fit this model to the averaged movie curves of Figure 4, representing SRSF1 protein levels with and without feedback for both low and high induction levels. For the no-feedback case, the relevant model parameters include the transcription rates of the ectopic construct with low $\left({\text{α}}_{ec}^{lo}\right)$ or high $\left({\text{α}}_{ec}^{hi}\right)$ induction, the translation rate (γ), and the degradation rates of mRNA (β_m_) and protein (β_p_). Since we measure only the ectopic SRSF1 protein, we do not need to model the endogenous SRSF1 here.

Once we introduce regulation at the level of splicing, we must consider the ectopic as well as the endogenous SRSF1. We also include the parameters describing the feedback: *k*, the ratio of full transcript spliced to the productive isoform; *k*, the activation coefficient (substrate concentration of half-maximum activity); *h*, the Hill coefficient; and *R*, the reservoir level.

In addition to these key model parameters, we fit the starting levels of endogenous SRSF1 species u_0_, p_0_, and m_0_. Based on the analysis of Figure 4, we account for time delays between induction and cellular response, considering two values t_low_ and t_high_ for the low- and high-induction conditions. We also account for background noise by setting a floor *bg* on the predicted output.

#### Parameter fitting

To determine the model parameters, we performed Bayesian inference using Stan (Carpenter et al., 2017) to sample the probability distributions for each parameter given the experimental data. Bayesian statistics requires specifying priors, or the probability distributions for each parameter based on prior knowledge, and the likelihood, or the probability of observing the data given a set of parameters. Together, they allow inference of the posterior, or the probability distributions for each parameter after observing the experimental data.

For the likelihood, we assumed that experimental values lie in a Gaussian distribution centered at the theoretical value with a standard deviation σ. For the priors, we assumed relatively broad distributions based on biological knowledge and included applicable constraints. These prior distributions for each parameter are summarized in Table S2. All parameters are constrained to be nonnegative; any additional constraints are listed.

For these differential equations, the starting levels of each species can be fit, but the starting time is not permitted to vary in this framework. Therefore, we screened a broad range of choices of time delays, performed an initial fit for all possible combinations, and selected the values that minimized the resulting error. These initial fits were done on 4 chains with 250 iterations per chain (125 warm up, 125 sampling). For time delays, we evaluated a range of 6–9 h in the low-induction case and a range of 0–1 h in the high-induction case, sampling the ranges at intervals of 0.25 h (15 min). To quantify error for each set of time delays, we took the median value across samples for each parameter, simulated the resulting curve, and calculated the sum of squared errors (SSE) between the theoretical and observed values. This procedure yielded an optimal set of time delays of 8.25 h for the low-induction condition and 0.25 h for the high-induction condition. We then reran a more extensive fitting for this set of values, using 4 chains with 2000 iterations per chain (1000 warm up, 1000 sampling). The resulting samples are the basis for all results presented in the main text.

## Supplementary Material

1

## Figures and Tables

**KEY RESOURCES TABLE:** 

REAGENT or RESOURCE	SOURCE	IDENTIFIER

Chemicals, peptides, and recombinant proteins		

Human Fibronectin	Oxford Biomedical Research	SKU:GP40
4-epi tetracycline hydrochloride	Sigma Aldrich	37918
doxycycline	Ding et al., 2019	N/A

Critical commercial assays		

Lipofectamine LTX plasmid transfection reagent	ThermoFisher	A12621
RNeasy Mini Kit	Qiagen	74106
iScript cDNA Synthesis Kit	Bio-Rad Laboratories	1708891
AccuPrime™ PfxSuperMix	ThermoFisher	12344–040
iQ SYBR Green Supermix	Bio-Rad Laboratories	1708880

Experimental models: Cell lines		

Flp-In™ T-Rex™ HEK293 cells	ThermoFisher	R78007
gDNA cells: FLP-in T-Tex 293 + CMV-TO- Citrine-EndoSFRS1	This paper	N/A
cDNA cells: FLP-in T-Tex 293 + CMV-TO- Citrine-cDNA of SFRS1	This paper	N/A
synT cells: FLP-in T-Tex 293 + CMV-TO- H2B-Ceru-3'UTR	This paper	N/A

Oligonucleotides		

RT-PCR *SRSF1* isoforms forward -ACATC GACCTCAAGAATCGCCGC	IDT DNA	N/A
RT-PCR *SRSF1* isoforms reverse -ATCCA GTGAGCCCTCTCCAA	IDT DNA	N/A
RT-PCR *SynT* isoforms forward- CACATG AAGCAGCACGACTT	IDT DNA	N/A
RT-PCR *SynT* isoforms reverse - ATCCA GTGAGCCCTCTCCAA	IDT DNA	N/A
*SRSF1* isoform1 gene-specific primer-TCA TCCTCCCTATCCTATCCACA	IDT DNA	N/A
RT-qPCR *SRSF1* isoform1 forward - GCA GAGGATCACCACGCTAT	IDT DNA	N/A
RT-qPCR *SRSF1* isoform1 reverse - GCC AAGGTTTAAAAAGCAAAGCA	IDT DNA	N/A
RT-qPCR *SynT* isoform 1 forward - CGG CATGGACGAGCTGTA	IDT DNA	N/A
RT-qPCR *SynT* isoform 1 reverse - AGTTC ACACAAACCAGGGCA	IDT DNA	N/A
*GAPDH* isoform1 gene-specific primer - AGT GATGGCAT GGACTGTGG	IDT DNA	N/A
RT-qPCR *GAPDH* forward - GGTGTGAACC ATGAGAAGTATGA	IDT DNA	N/A
RT-qPCR *GAPDH* reverse - GAGTCCTTCC ACGATACCAAAG	IDT DNA	N/A
*SDHA* isoform1 gene-specific primer - CTC CAGTGCTCCTCAAAGGG	IDT DNA	N/A
RT-qPCR *SDHA* forward - AGAGGGAGGC ATTCTCATTAAC	IDT DNA	N/A
RT-qPCR *SDHA* reverse - ACCGAGACAC CACATCTCTA	IDT DNA	N/A

Recombinant DNA		

SRSF1(gDNA): CMV-TO-Citrine-EndoSFRS1	This paper	N/A
SRSF1(cDNA): CMV-TO-Citrine-cDNA of SFRS1	This paper	N/A
SynT: CMV-TO-H2B-Ceru-3’UTR	This paper	N/A

Software and algorithms		

Automated acquisition software Metamorph	Molecular Devices	N/A
Easyflow	Y. Antebi	https://github.com/AntebiLab/easyflow
Fiji	Schindelin et al., 2012	https://imagej.net/Fiji
Cell tracking	Y. Antebi	https://github.com/AntebiLab/EasyTrack
MATLAB	MathWorks	N/A
The code and database for tissue-specific analysis (related to Figures 6 and S8–S10)	This paper	https://doi.org/10.5281/zenodo.6522983
The code for the mathematical modeling section (related to Figures 7 and S11)	This paper	https://doi.org/10.5281/zenodo.6525681
The deep-learning image analysis code	Darvin Yi	https://github.com/yidarvin/FirstAid

Other		

24-well 10 mm diameter glass No. 1.5 coverslip plates	MatTek Corp	P24G-1.5–10-F

## Highlights

- SRSF1 uses negative autoregulatory splicing to maintain its own concentration
- This feedback reduces heterogeneity in both SRSF1 levels and response times
- Splice autoregulation adapts SRSF1 levels to variation in target transcript load
- A minimal mathematical model explains these capabilities
