# Supplementary material for: Dynamics and functional roles of splicing factor autoregulation
Source: Cell Rep. Author manuscript; Available in PMC 2022 Jul 7. (PMC9262138; doi:10.1016/j.celrep.2022.110985)
Supplement: 1 [file NIHMS1818171-supplement-1.pdf]

Cell Reports, Volume *39*

Supplemental information

Dynamics and functional roles

# of splicing factor autoregulation

Fangyuan Ding, Christina J. Su, KeHuan Kuo Edmonds, Guohao Liang, and Michael B. Elowitz

Supplementary Information

Dynamics and functional roles of splicing factor autoregulation

Fangyuan Ding, Christina J. Su, KeHuan Kuo Edmonds, Guohao Liang, Michael B. Elowitz

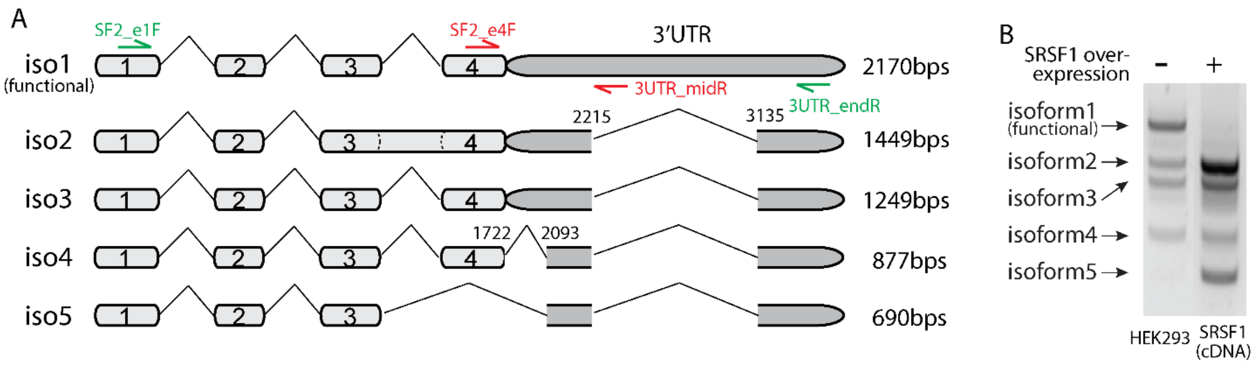

Figure S1: 5 isoforms of *SRSF1* and Citrine-fused *SRSF1* were observed in HEK293 cells. Related to Figure 1 and 2. **A**) We obtained the sequences of 5 isoforms by Laragen@ sequencing after RT-PCR (STAR Methods) and gel extraction. Labeled primers are for RT-PCR (green) and RT-qPCR (red) respectively (Table S1). **B**) SRSF1 overexpression promotes unproductive splicing of its own gene. We used RT-PCR and gel-imaged 5 isoforms of *SRSF1* of HEK293 cells (left lane) and of *SRSF1*(cDNA) cells with maximum induction level (100ng/ml dox) for 50hrs (right lane). We found that SRSF1 overexpression reduced the ratio of isoform 1 (i.e. the functional isoform that can be productively translated to SRSF1(Sun et al., 2010)), and increased expression of the other 4 unproductive isoforms.

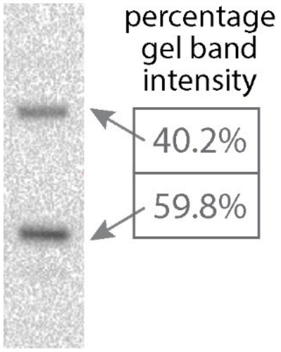

Figure S2: Western blot gel band intensity quantification shows that the endogenous SRSF1 protein level is higher than the ectopic copy. **Related to Figure 2B**. We analyzed the gel band intensity (gDNA version in Figure 2B with highest induction level) using a Bio-Rad Chemi-Doc Image Lab 6.0 band analyzer.

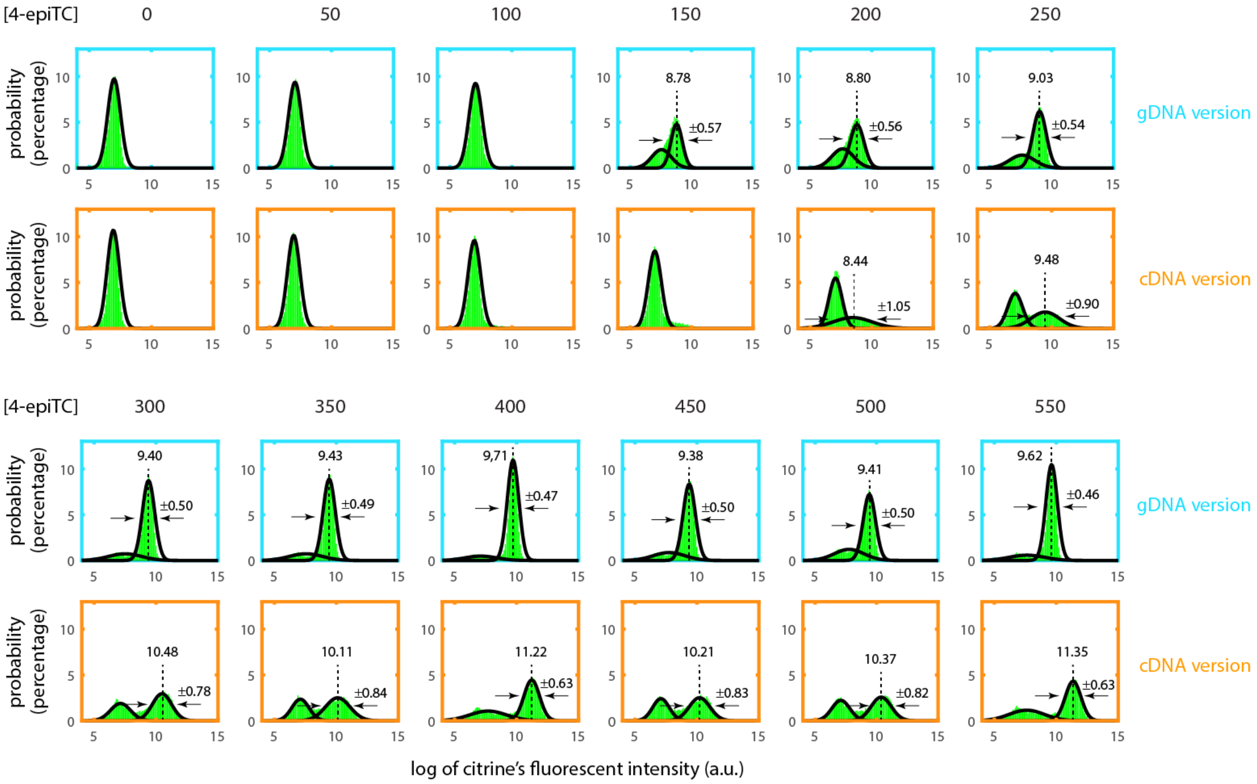

Figure S3: Flow cytometry data were analyzed by Gaussian fitting. Related to Figure 2 and 4. One typical example of experimental replicates is shown here. The apparent bimodal distributions with short induction time (<24hrs) are probably due to cell-cell heterogeneity in 4-epiTC absorption efficiency, and stochastic transcriptional noise from the CMV promoter. This bimodality diminishes for longer induction time. To minimize the impact of this bimodal effect, we used the mean of Gaussian fitting from only fully induced cells (i.e. high peak) to represent SRSF1 expression in Figure 2C. The Gaussian fitting center and variance are labeled in each plot.

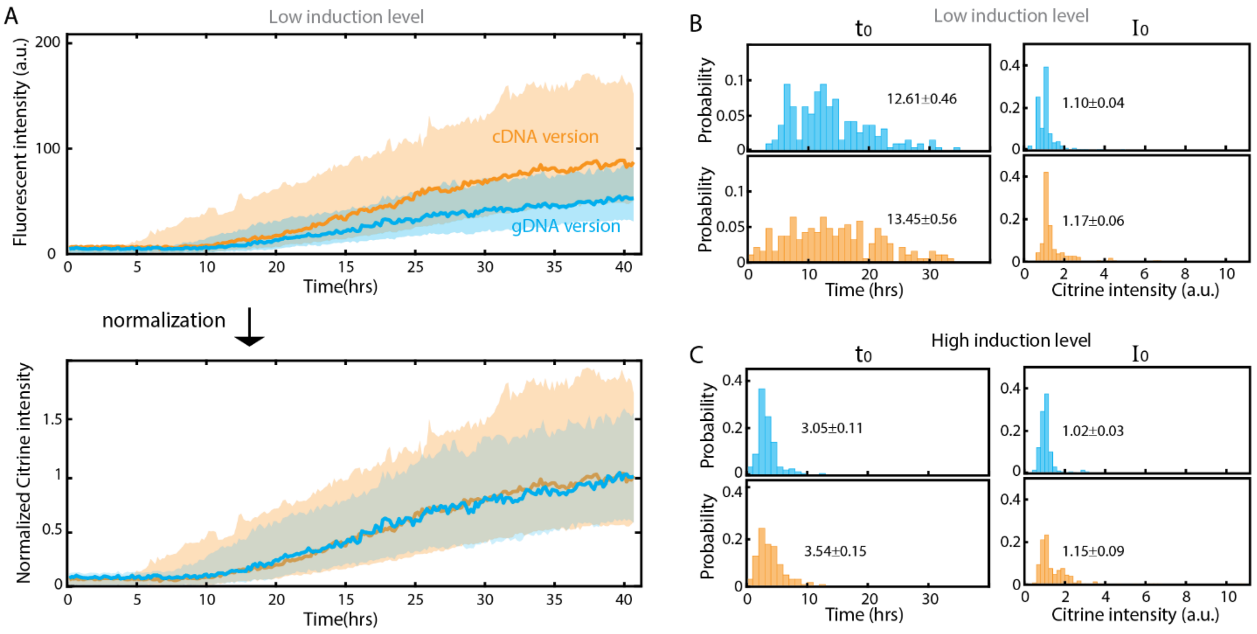

Figure S4: Negative autoregulatory splicing reduces cell-cell heterogeneity in both response rate and steady-state SRSF1 expression at low induction level. **Related to Figure 4**. **A**) (Top) Solid curves are the median of 191 *SRSF1*(cDNA) and 188 *SRSF1*(gDNA) single cell traces. Shading represents the standard deviation of the mean. (Bottom) The curves are normalized to final expression. **B**) and **C**) are distribution of fit-parameters t0 and I0 defined in Figure 4C. Color is the same as in Figure 4D and 4E.

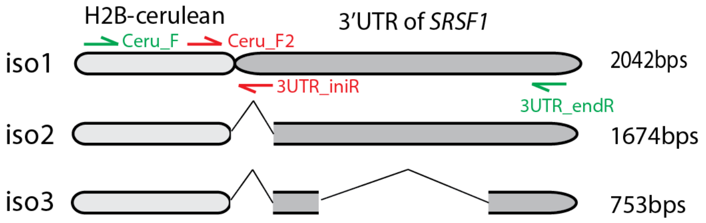

Figure S5: 3 isoforms of *SynT* were observed in HEK293 cells. **Related to Figure 5**. We sequenced each isoform using Laragen@ sequencing after RT-PCR (STAR Methods). Labeled primers are for RT-PCR (green) and RT-qPCR (red) respectively (Table S1).

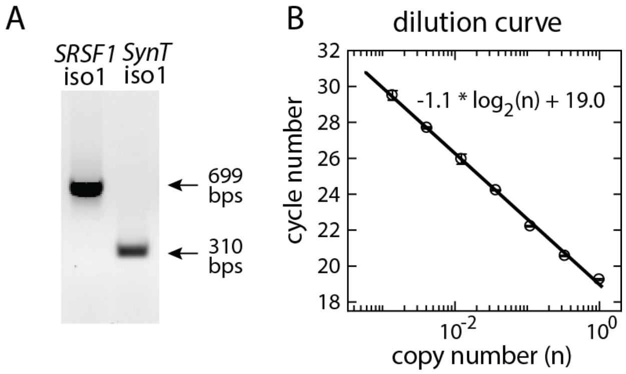

Figure S6: Quality control of RT-qPCR. **Related to Figure 5**. **A**) The products of *SRSF1* isoform 1 and *SynT* isoform 1 RT-qPCR are 699bps and 310bps respectively. The single band indicates the specificity of qPCR amplification. **B**) The unusual 699bps length qPCR still obeys linear rules in the dilution curve.

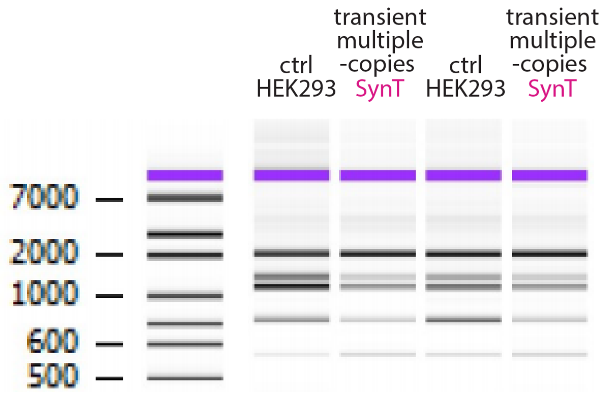

SRSF1 isoforms percentage

iso1	27.8%	46.0%	32.2%	44.9%
iso2	22.3%	12.4%	16.5%	13.2%
iso3	30.8%	23.2%	23.1%	22.2%
iso4	16.7%	11.0%	24.1%	12.4%
iso5	2.5%	7.3%	4.1%	7.3%

Figure S7: **The amount of SRSF1 isoform1 responds to splicing 'load'. Related to Figure 5E**. When SynT level increased (via transiently transfect *SynT* for 5.5hrs), a higher fraction of SRSF1 pre-mRNA remained unspliced, producing more functional isoform 1. The listed concentration quantification is achieved via Agilent Bioanalyzer 2100 in UCI Genomics High Throughput Facility.

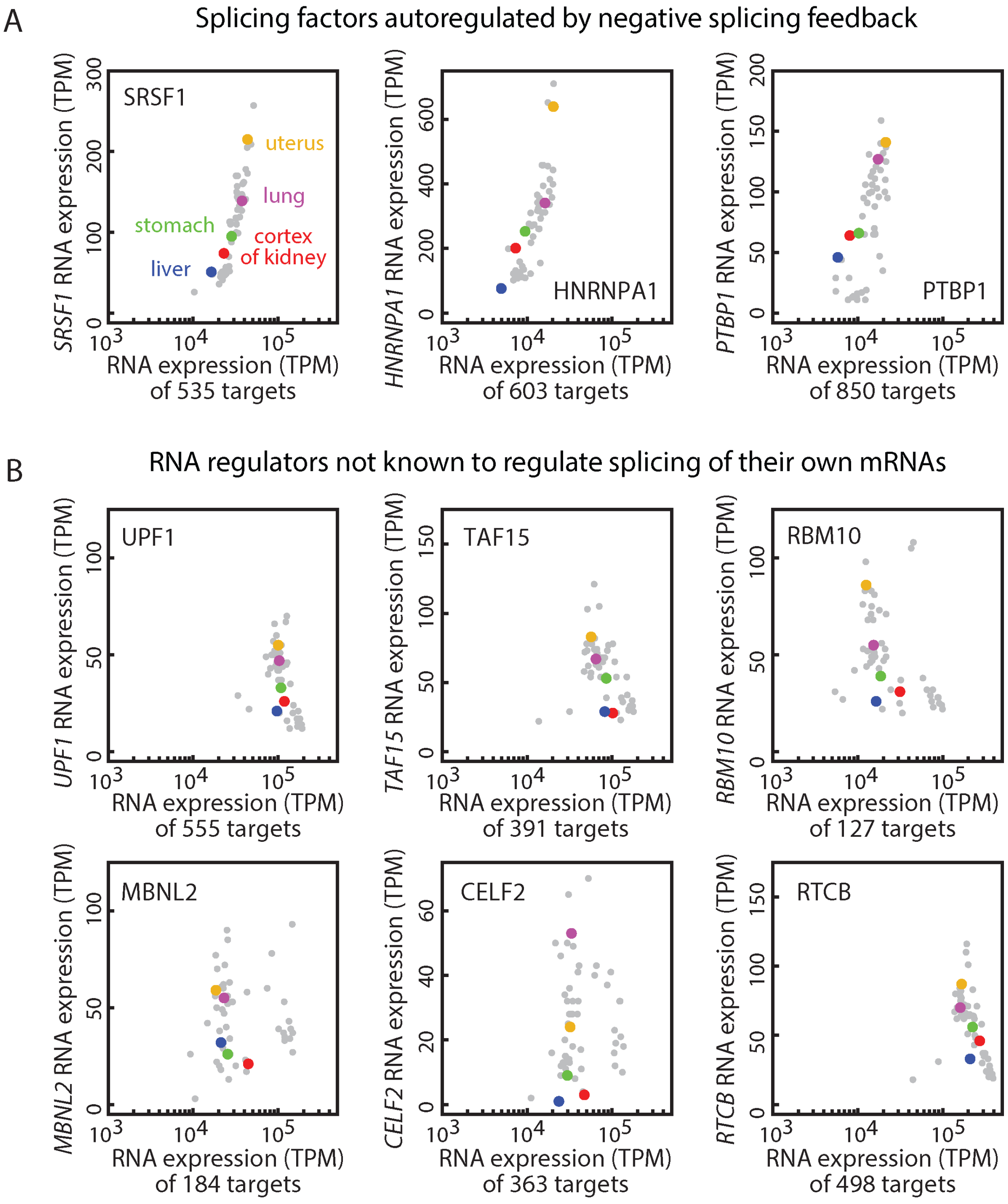

Figure S8: **Relationships between splice factors and target expression are preserved with 5 percentile criteria for target gene selection. Related to Figure 6**. All data and labels are similar to those in Figure 6, except in this case a distinct criterion for target gene selection was used. Here, target genes were required to appear in the top 5% of CLIP-Seq "Binding site records" (more details in STAR Methods).

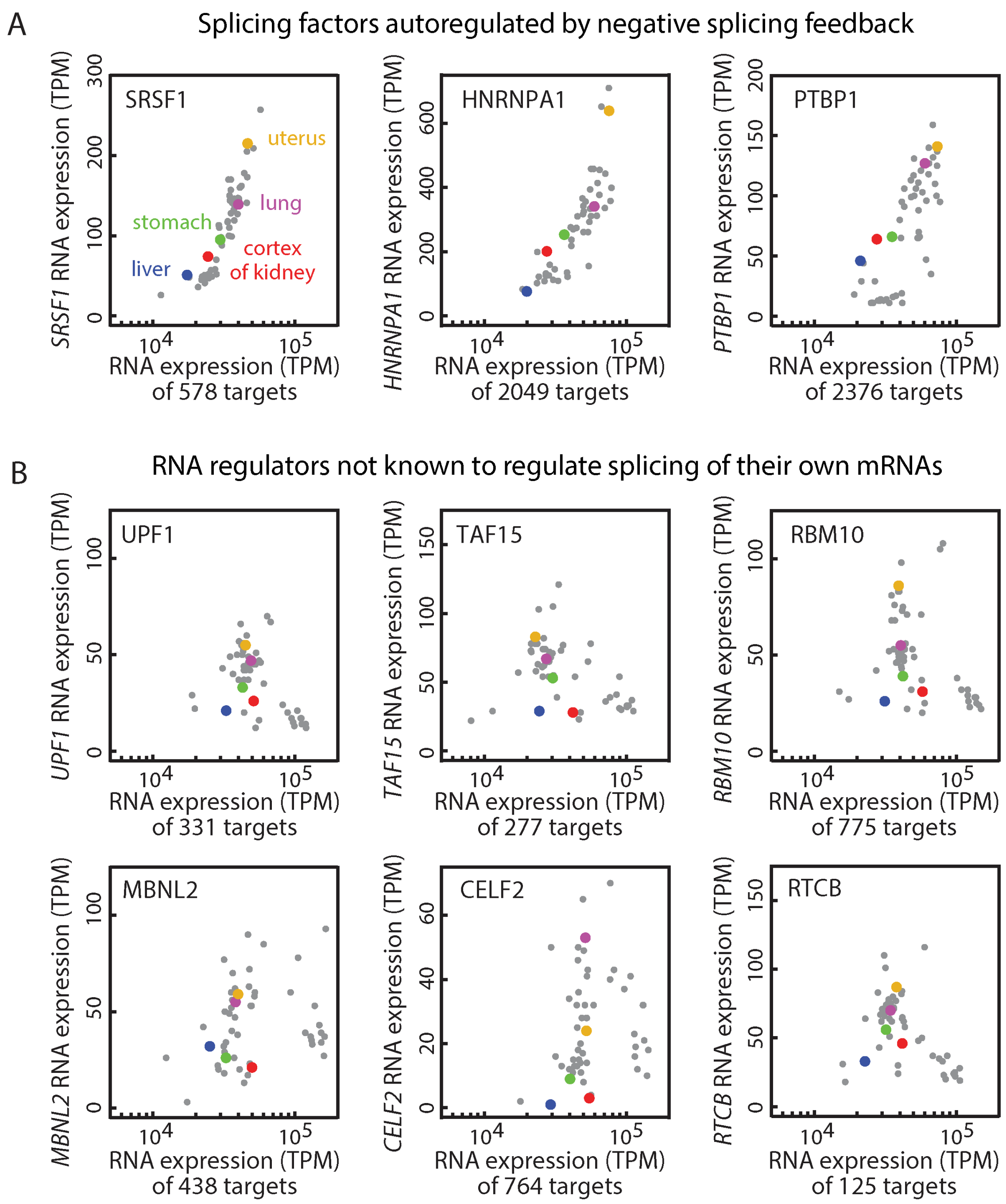

Figure S9: **Relationships between splice factors and target expression are preserved with absolute abundance criteria for target gene selection. Related to Figure 6**. All data and labels are similar to those in Figure 6, except in this case a distinct criterion for target gene selection was used. Here, target genes were identified based on an absolute threshold of “Binding site records” (more details in STAR Methods).

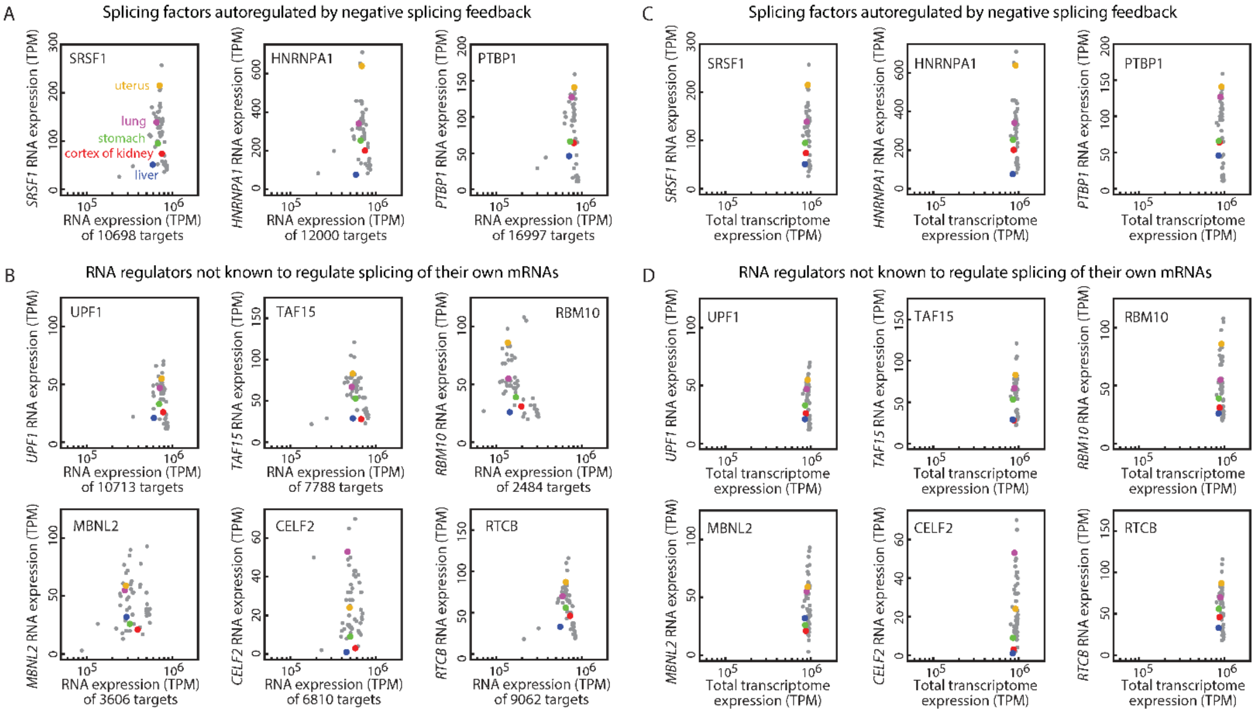

Figure S10: **Control read-out of TPM**. **Related to Figure 6, S8 and S9**. **A, B**) Summing up TPM of all CLIP-seq marked genes. The result is closed to total transcriptome reads, indicating the needs to set up a threshold (more details in STAR Methods). **C, D**) Summing up TPM of all genes (i.e. total transcriptome) from the GTEx dataset (www.gtexportal.org) shows a constant value (~10^6), indicating the value is properly normalized across different tissue types.

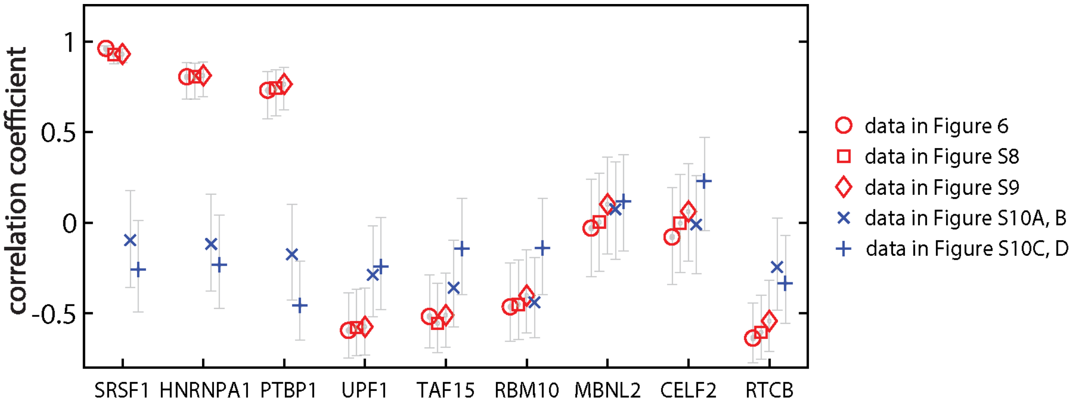

Figure S11: **Correlation coefficient of data from Figure 6, S8, S9 and S10**. Correlation coefficients between splice regulators and other genes. The result confirms the three negative regulated splicing factors, SRSF1, hnRNPA1, and PTBP1, shows the feedback adaptation to their target level, while the other 6 not. Error bars are the 95% confidence intervals.

A

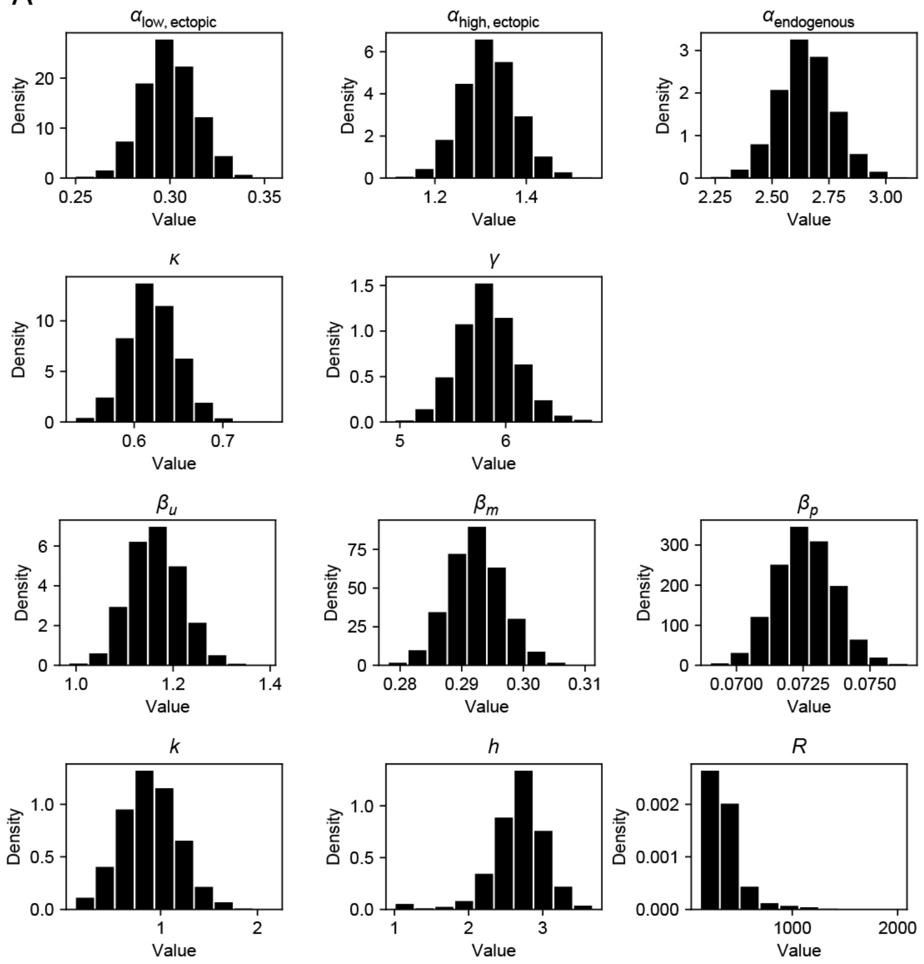

B

	median	95% interval
σ	5.57	(5.30, 5.87)
u_0	0.66	(0.27, 1.04)
m_0	0.05	(0.00, 0.27)
p_0	1.93	(0.07, 8.81)
backgrond	5.69	(4.91, 6.46)

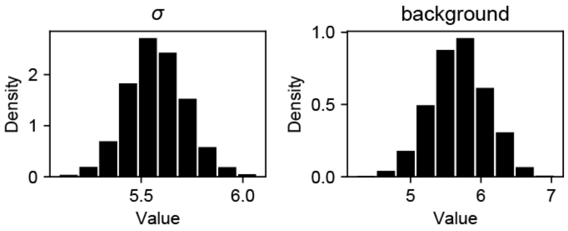

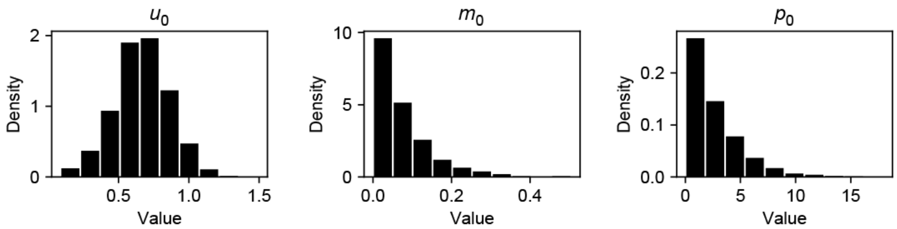

Figure S12: **The histogram of parameters obtained from minimal mathematical model fitting**. **Related to Figure 7**. **A**) Distribution of all parameters shown in Figure 7B Table. **B**) The fitting estimates and distribution of all other parameters.

Table S1: The sequence of all primers used in this paper. Related to Figure 5, S1, S5, and S6.

Purpose of the primer	Primer name	Sequence
RT-PCR *SRSF1* isoforms	SF2_e1F	ACATCGACCTCAAGAATCGCCGC
RT-PCR *SRSF1* isoforms	3UTR_endR	ATCCAGTGAGCCCTCTCCAA
RT-PCR *SynT* isoforms	Ceru_F	CACATGAAGCAGCACGACTT
RT-PCR *SynT* isoforms	3UTR_endR	ATCCAGTGAGCCCTCTCCAA
SRSF1 isoform1 gene-specific primer	3UTR_RT	TCATCCTCCCTATCCTATCCACA
RT-qPCR *SRSF1* isoform1	SF2_e4F	GCAGAGGATCACCACGCTAT
RT-qPCR *SRSF1* isoform1	3UTR_midR	GCCAAGGTTTAAAAAGCAAAGCA
SynT isoform1 gene-specific primer	3UTR_RT	TCATCCTCCCTATCCTATCCACA
RT-qPCR *SynT* isoform 1	Ceru_F2	CGGCATGGACGAGCTGTA
RT-qPCR *SynT* isoform 1	3UTR_iniR	AGTTCACACAAACCAGGGCA
GAPDH isoform1 gene-specific primer	GAPDH_RT	AGTGATGGCATGGACTGTGG
RT-qPCR *GAPDH*	GAPDH_F	GGTGTGAACCATGAGAAGTATGA
RT-qPCR *GAPDH*	GAPDH_R	GAGTCCTTCCACGATACCAAAG
SDHA isoform1 gene-specific primer	SDHA_RT	CTCCAGTGCTCCTCAAAGGG
RT-qPCR *SDHA*	SDHA_F	AGAGGGAGGCATTCTCATTAAC
RT-qPCR *SDHA*	SDHA_R	ACCGAGACACCACATCTCTA

Table S2: The parameter constraints and initial value distributions of our minimal mathematical model fitting. Related to Figure 7 and S12.

Parameter	Distribution	Constraints
$\alpha_{\text{low, ec}}$	Norm(1, 0.2)	
$\alpha_{\text{high, ec}}$	Norm(5, 1)	$2\alpha_{\text{low, ec}} < \alpha_{\text{high, ec}}$
α_{en}	Norm(1, 0.2)	$2\alpha_{\text{high, ec}} < \alpha_{\text{en}} < 4\alpha_{\text{high, ec}}$
κ	Norm(2, 0.5)	
γ	Norm(2, 0.5)	
β_u	Norm(0.4, 0.08)	
β_m	Norm(0.2, 0.02)	
β_p	LogNorm(-6, 1)	$\beta_p < \beta_m/4$
k	Norm(1, 0.3)	
h	Norm(2, 0.5)	$1 < h$
R	LogNorm(7, 1)	
σ	Norm(10, 2.5)	
u_0	Norm(1, 0.2)	
m_0	Norm(3, 1)	
p_0	Norm(50, 10)	
bg	Norm(5, 1)	

Norm(u, sigma): normal distribution with mean mu and standard deviation sigma
LogNorm(mu, sigma): log-normal distribution with mean mu and standard deviation sigma
